# Novel roles of LSECtin in gastric cancer cell adhesion, migration, invasion, and lymphatic metastasis

**DOI:** 10.1038/s41419-022-05026-x

**Published:** 2022-07-11

**Authors:** Yinan Zhang, Zhen Feng, Yue Xu, Sufen Jiang, Qianshi Zhang, Zhenyu Zhang, Keyong Wang, Xiaomeng Li, Lijie Xu, Menglang Yuan, Zihao Chen, Jingyi Cui, Han Wu, Yina Gao, Wei Wei, Bo Wang, Yunfei Zuo, Shuangyi Ren

**Affiliations:** 1 Department of General Surgery, The Second Hospital of Dalian Medical University, 116023 Dalian, China; 2 Department of Clinical Biochemistry, College of Laboratory Medicine, Dalian Medical University, 116044 Dalian, China; 3 Department of Functional Laboratory, College of Laboratory Medicine, Dalian Medical University, 116044 Dalian, China; 4 Department of Pathology and Forensics, Dalian Medical University, Dalian, 116044 China

**Keywords:** Cancer, Cell adhesion, Cell migration, Non-coding RNAs

## Abstract

Liver and lymph node sinusoidal endothelial cell C-type lectin (LSECtin) plays an important regulatory role in a variety of diseases, including tumors. However, the underlying mechanism of LSECtin in gastric cancer (GC) remains largely unknown. In our research, LSECtin promoted the adhesion and invasion of GC cells, and was involved in lymphatic metastasis of GC cells. Mechanistically, LSECtin promoted the adhesion, proliferation and migration of GC cells by downregulating STAT1 expression. The circular RNA circFBXL4, which is regulated by LSECtin, sponges the microRNA miR-146a-5p to regulate STAT1 expression. The promotion of GC cell proliferation, migration and invasion mediated by LSECtin was largely inhibited by circFBXL4 overexpression or miR-146a-5p silencing. Moreover, in its role as a transcription factor, STAT1 modulated the expression of FN1 and CHD4. In conclusion, LSECtin might be involved in the lymphatic metastasis of GC by upregulating the expression of FN1 and CHD4 via the circFBXL4/miR-146a-5p/STAT1 axis, possibly indicating a newly discovered pathogenic mechanism.

## Introduction

Gastric cancer (GC) is a leading cause of mortality worldwide [1]. Although surgical resection combined with adjuvant therapies is an established treatment, GC with invasion and distant metastasis leads to poor prognosis and a low survival rate [2]. It is therefore of great importance to have a better understanding of the largely unknown molecular mechanisms underlying GC growth and progression.

Liver and lymph node sinusoidal endothelial cell C-type lectin (LSECtin) is a 40 kDa type II transmembrane protein, that in the C-type lectin DC-SIGN family, which also includes dendritic cell-specific intercellular adhesion molecule 3 capturing nonintegrins (DC-SIGN), DC-SIGN-related proteins (DC-SIGNR), and CD23 [3, 4]. The expression of LSECtin is mainly found in sinusoidal endothelial cells of the human liver and lymph nodes with DC-SIGN expression [3]. The DC-SIGN family has a C-terminal calcium-dependent carbohydrate recognition domain (CRD) [3], whose elevated expression usually accompanied with cancer progression [5]. LSECtin enhances breast cancer stemness via interaction with its receptor BTN3A3 [6]. Our previous paper reported the participation of the DC-SIGN family proteins in carcinogenesis and cancer metastasis of gastrointestinal tumors. In serum from colon cancer patients, the expression of LSECtin and DC-SIGNR was found to be elevated, and in cases of liver metastasis, the expression was further increased [7, 8], as was that of DC-SIGNR in GC [9]. DC-SIGN and DC-SIGNR can affect the expression of long noncoding RNA (lncRNA) by upregulating STAT3 and STAT5A expression to promote the progression and metastasis of GC [9, 10]. In summary, it is reasonable to hypothesize that LSECtin participates in tumor progression.

Circular RNAs (CircRNAs) are endogenous noncoding RNAs with closed-loop structure produced by pre-mRNA through variable shear processing [11]. The expression and functions of circRNAs are stable, abundant, conserved, and highly tissue-specific, serving as key participants in many different cancers [12–14]. Some studies have reported that circRNAs engage in microRNA (miRNA) molecular spongeing by sequestering miRNAs and inhibiting their interactions with target mRNAs, and through this process, they can play regulatory roles in various diseases [15]. cSMARCA5 inhibits the growth and metastasis of HCC in vitro and in vivo by sponging miR-17-3p and miR-181b-5p to upregulate TIMP3 [16]. It has been validated that circNRIP1 is a tumor promotor in GC that sponges miR-149-5p to affect the expression level of AKT1 [17]. This evidence indicates roles for circRNAs in the carcinogenesis of different cancers.

In our study, we aimed to investigate the mechanism of LSECtin-induced proliferation, invasion, and migration of GC cells in vitro. Our data indicated that LSECtin downregulates the expression of STAT1 through the circFBXL4/miR-146a-5p axis, which ultimately leads to increased expression of FN1/CHD4 resulting in an increased risk of cancer. Our findings provide new insights into the molecular mechanisms of GC progression.

## Materials and methods

### Microarray analysis

MGC-803 cells were incubated with the recombinant LSECtin protein or control IgG for 24 h, and total RNA was isolated and analyzed with a Human RT2 Profiler TM PCR Array (KangChen Biotech Company, shanghai, China), which selected 84 genes related to tumorigenesis. Data normalization was performed based on the average Ct values from several housekeeping genes and differentially expressed genes were identified. Total RNA of 3 pairs of GC samples (tumor and counterpart nontumor tissues) was extracted and processed according to the circular RNA microarray manufacturer’s standard protocols. The Arraystar Human circRNA Array v2 (8x15K, Arraystar) hybridization and collection of data were performed by KangChen Biotech. The differentially expressed circRNAs had a fold change ≥2.0 and *p* < 0.05. GC tissues confirmed by pathologic examination were obtained from the Second Affiliated Hospital of Dalian Medical University. Experimental procedures were approved by the ethics committee of the Second Affiliated Hospital of Dalian Medical University. Informed consent was obtained from all patients.

### Cell culture and treatments

A human gastric mucosal epithelial cell line (GES-1) was purchased from the Xiangya Experiment Center (Changsha, China), Human GC cells (AGS, BGC-823, MGC-803, and SGC-7901) and HEK293 cell lines were purchased from the Institute of Biochemistry and Cell Biology of the Chinese Academy of Sciences (Beijing, China). AGS, BGC-823, MGC-803, and SGC-7901 cells were cultured in RPMI 1640 medium (Gibco, California, USA), GES-1 and HEK293 cells were cultured in DMEM (Gibco) with 10% fetal bovine serum (BD Bioscience, San Jose, CA, USA or ExCell Bio, Shanghai, China), at 37 °C in a humidified incubator with 5% CO_2_. Culture flasks were purchased from JET BIOFIL (Guangzhou, China). Cells were treated with 2 mg/ml actinomycin D (Meilune, Dalian, China) for 4, 8, 12, and 24 h, with dimethylsulfoxide as a negative control. LSECtin or STAT1 overexpression plasmids, short interfering RNAs (siRNAs) targeting STAT1 or circFBXL4, and miR146a-5p mimic and inhibitor were purchased from GenePharma (Shanghai, China) and then were transfected with Lipofectamine 2000 (Invitrogen, Carlsbad, CA). The sequences and NCBI identifiers are available as Additional files 1 and 2. Cells were treated with the STAT1 activation inhibitor fludarabine (5 µM, 24 h) (MedChemExpress, Dalian, China), with dimethylsulfoxide as a negative control.

### Cell adhesion and flow cytometry

After treated with recombinant human LSECtin or control IgG on ice for 2 h and then incubated with an anti-LSECtin goat monoclonal antibody (1:100, Sigma, St.Louis, USA) on ice for 1 h, the cells were incubated with FITC-labeled rabbit anti-goat IgG secondary antibody (1:200, ZSGB-BIO, Beijing, China) for another 1 h, and then washed and fixed in 4% paraformaldehyde. The adherent cells were analyzed through flow cytometry (FACScan; BD Biosciences) equipped with Summit 5.0 software. Alternatively, frozen lymph node sections were incubated with GC cells and then placed on a plate shaker at low speed for 40 minutes after blocking in goat anti-mouse LSECtin polyclonal antibody (Abcam, Cambridge, USA) or IgG (ZSGB-BIO) at a 1:50 dilution. The fixed adherent cells were H&E stained and counted using ImageJ software. The schematic overview of the adhesion experiment is shown in Supplementary file 7 Figure S1A.

### Quantitative real-time PCR (qPCR) and Western blot analysis

Details can be found in the supplementary Materials and methods section.

### ELISA

The serum levels of LSECtin were measured and analyzed with an ELISA kit (Lengton, Shanghai, China). ELISA was carried out in accordance with manufacturer’s instructions.

### In-vitro proliferation, migration and invasion assays

Details can be found in the supplementary Materials and methods section.

### Chromatin immunoprecipitation assay (ChIP)

ChIP was performed using an EZ-Magna ChIP assay kit (Merck Millipore, Billerica, MA, USA) according to manufacturer’s instructions. BGC-823 (1 × 10^7^) and MGC-803 (1 × 10^7^) cells were incubated with 1 μg/mL recombinant LSECtin protein for 24 h, then crosslinked, lysed and sonicated. Lysates were immunoprecipitated with antibodies against STAT1(Cell Signaling Technology, Danvers, MA, USA), or nonspecific IgG. qRT-PCR was performed to amplify the purified DNA fragment (TIANGEN).

### RNA immunoprecipitation (RIP)

A RIP assay was carried out in accordance with manufacturer’s instructions of a RIP Kit (BersinBio, GuangZhou, China). Approximately 1 × 10^7^ BGC-823 cells were collected and incubated with RIP lysates, and then incubated with buffer containing magnetic beads conjugated with human anti-Ago2 antibody (Abcam, Cambridge, USA) or control IgG antibody. Immunoprecipitated RNA was retrotranscribed with random primers, and measured by qRT-PCR.

### Luciferase reporter

The wild-type or mutant miR-146a-5p binding sequence of circFBXL4 was amplified by PCR and cloned into a pmirGLO dual-luciferase vector (Promega, Madison, WI, USA). The miR-146a-5p mimic or negative control, and the pmirGLO dual-luciferase vector were cotransfected into HEK293 cells and cultured with 48 h. The ratio of firefly to Renilla luciferase activity was detected with a Dual Luciferase Reporter assay kit (Promega).

### RNA fluorescencein situ hybridization (FISH)

A FISH kit and probes were purchased from GenePharma. FISH assay was performed according to manufacturer’s instructions. Briefly, BGC-823 cells with the fixation of 4% paraformaldehyde were incubated with Cy3-labeled circFBXL4 probe and FAM-labeled miR-146a-5p probe overnight, stained with DAPI, and finally photographed by microscopy (Leica, Heidelberg, Germany).

### Animal studies

Ten 4-week-old BALB/c athymic nude mice were assigned randomly to two groups consisting of five mice each. BGC-823 cells (1 × 10^7^) incubated with 5 μg/mL recombinant LSECtin protein or isotype IgG for 2 h in 37 °C were injected into the footpads of nude mice. The schematic overview of the experiment is shown Supplementary file 7 Figure S1F. The animals were killed after 24 days, and the popliteal, inguinal and axillary lymph nodes were removed and embedded in paraffin, and hematoxylin and eosin (H&E) staining was performed todetermine the numbers of lymph node metastases. Approval and support for this experiment were obtained from the Animal Center of Dalian Medical University.

### Bioinformatics analysis

The Cancer Genome Atlas (TCGA) data provided by the Broad Institute’s GDAC Firehose can be accessed in the Bioconductor package RTCGAToolbox [18]. The expression profiles were downloaded as gdac.broadinstitute.org_STAD.mRNAseq_Preprocess. Level_3 and gdac.broadinstitute.org_STAD.miRseq_Preprocess.Level_3. RNA-seq data were normalized with RSEM software and reported as log2(Reads), representing the expression values. Survival analysis of the GC patients with target gene expression was performed using the Kaplan-Meier Plotter website. Database details can be found in Supplementary file Table S3. Gene Ontology (GO) and Kyoto Encylopedia of Genes and Genomes (KEGG) pathway analyses performed by KangChen Biotech Company.

### Statistical analysis

All experiments were repeated at least three times, and the data are presented as the mean ± SD. ANOVA (multiple groups) and t-tests (two groups) were performed to analyze the data using GraphPad Prism 7.0. *P* value of 0.05 or less was considered to be statistically significant.

## Results

### LSECtin is increased in GC serum and correlates with GC lymph node metastasis

**Fig. 1:**
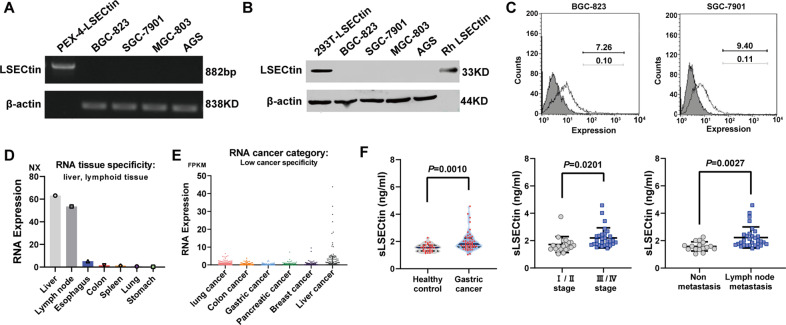
LSECtin is increased in GC serum and correlated with the occurrence of GC. **A**, **B** Expression of LSECtin in GC cells. PEX-4-LSECtin (plasmid) was choosen as a positive control for RT-PCR, with 293T-LSECtin, and Rh LSECtin serving as positive controls in Western blot (*n* = 3). **C** Cell surface level of LSECtin in GC cell lines was examined by flow cytometry. The numbers listed are percentage of positive cells. Black line, LSECtin; Gray line, isotype control. **D**, **E** The expression of LSECtin in normal tissues. **F** Soluble LSECtin (sLSECtin) levels in serum derived from GC patients. Data, the means ± SD.

To clarify the role of LSECtin in GC, the expression of LSECtin was investigated in GC cells by PCR, Western blot, and flow cytometry (Fig. 1A–C). No or limited expression of LSECtin was found in the 4 GC cell lines. Additionally, The Human Protein Atlas database showed LSECtin expressed at a high level in the liver and lymph nodes but low or no expression in the stomach or GC tissue (Fig. 1D, E). In this study, the data obtained for 78 participants GC patients (*n* = 48) and healthy controls (*n* = 30), were investigated, and serum LSECtin (sLSECtin) levels were quantified via ELISAs. Interestingly, there was a significant positive correlation between sLSECtin levels and GC. Moreover, a significant difference in sLSECtin levels between stage III/IV and I/II was observed. Additionally, the sLSECtin concentrations were significantly higher in GC patients with lymph node metastasis than in patients without metastasis (Fig. 1F). Interestingly, although LSECtin was rarely expressed in gastric cancer, These results sLSECtin was related to the process of lymph node metastasis of GC, which is major concern. According to a literature review, LSECtin expressed on the surface of the liver is involved in colon cancer liver metastasis [8]. Thus it is likely that LSECtin expressed on the surface of lymphatic sinus endothelial cells (LSECs), but not the surface of GC cells, promotes metastasis of GC cells. Further functional experiments are needed to prove this hypothesis.

### LSECtin positively regulates GC cell adhesion, proliferation, migration, and invasion in vitro and lymphatic metastasis in vivo

**Fig. 2:**
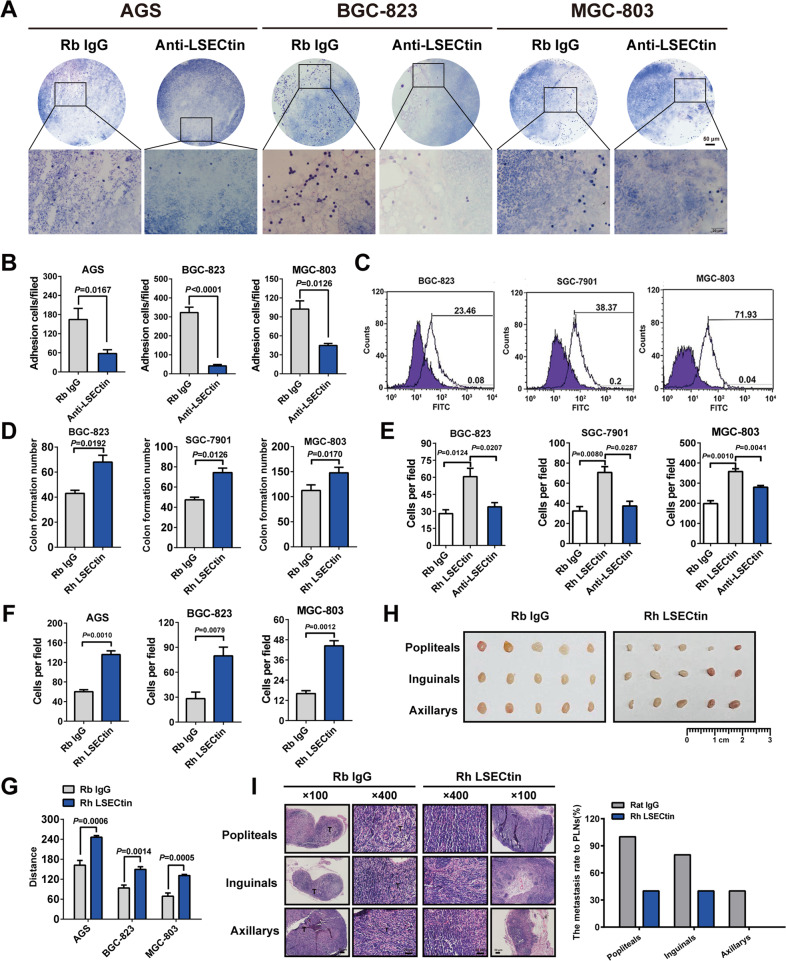
LSECtin positively regulates GC cell adhesion, proliferation, migration, and invasion in vitro and lymphatic metastasis in vivo. **A**, **B** Adhesion in vitro between GC cells and frozen mouse lymph node sections was detected by H&E staining (A). Histogram shows quantification analysis of cell number (B). **C** Adhesion of LSECtin to several GC cells was detected by flow cytometry. **D** Quantification of the clonogenic assay results after cells were treated with the 5 μg/ml LSECtin protein or control IgG. **E**, **F** The migratory and invasive abilities of GC cells treated with the 5 μg/ml LSECtin protein or control IgG were determined by Transwell assay. **G** The results of wound healing assays after cells were treated with the 5 μg/ml LSECtin protein or control IgG. **H** Pictures of lymph nodes harvested from nude mice 24 days after injected. **I** Representative H&E staining showing metastatic nodules and metastatsis rates. Data, the means ± SD (*n* = 3).

The adhesion experiment results were shown in Fig. 2A–B and S1A, a binding was observed between mouse LSECtin expressed in lymph nodes and human GC cells. This expression was particularly salient in the lymphoid sinus, where LSECtin was primarily expressed. However, binding was diminished by application of a LSECtin-blocking antibody. Flow cytometry results showed the binding between LSECtin and GC cells (Fig. 2C). To investigate the biological function of LSECtin in GC progression, we conducted a colony formation assay and found a significant difference in the number of colonies between LSECtin-treated and isotype IgG control group (Fig. 2D, S1B). The results of Transwell and wound healing assays indicated that LSECtin could promote the migration and invasion of GC cells; furthermore, GC cells migration was inhibited by a LSECtin-blocking antibody (Fig. 2E–G, S1C–E). We further investigated whether LSECtin is involved in lymphatic metastasis of GC. In the in vivo experiment, BGC-823 cells incubated with LSECtin protein in vitro inhibited binding of GC cells to LSECtin expressed on lymph nodes, injected into the footpads of nude mice, similar to binding competitive inhibition (Fig. S1F). As shown in Fig. 2H, S1G–I, the lymph node weight in the LSECtin treatment was lower than that in the control group, meanwhile the volume of popliteal lymph nodes was smaller than in the control group. The results revealed that LSECtin protein treatment in vitro blocked the adhesion of GC cells and LSECtin in lymph nodes and inhibited the lymphatic metastasis of GC (Fig. 2I). Thus, we concluded that LSECtin plays a potential role in the lymphatic metastasis of GC.

### STAT1 downregulation by LSECtin is involved in GC cell adhesion and migration

**Fig. 3:**
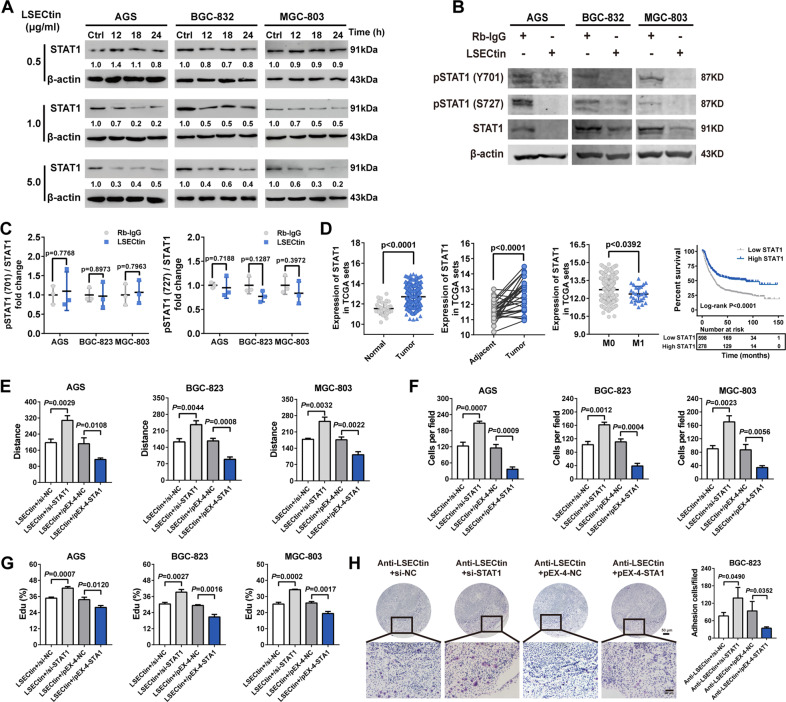
STAT1 downregulation by LSECtin is involved in GC cell adhesion and migration. **A** The expression of STAT1 in GC cells treated with 0.5, 1, and 5 μg/ml LSECtin protein and control IgG for 12 h was detected by Western blot analysis. **B**, **C** The expression of p-STAT1 and STAT1 protein in gastric cancer (GC) cells treated with 5 μg/ml LSECtin protein and control IgG for 24 h was detected by Western blot analysis (**B**). Semiquantitation of the phosphorylation level of STAT1 and STAT1 protein was from Western blot analysis (**C**). **D** STAT1 expression levels in 415 samples of GC and 35 samples of normal tissue, STAT1 expression levels in 32 tumor and matched normal samples from GC patients, STAT1 expression levels in M0 and M1 gastric tumors from TCGA were analyzed, and survival analysis curves were generated from 876 GC patients using the Kaplan-Meier plotter database. M0, no distant metastasis; M1, distant metastasis. **E** Wound healing assays of GC cells with LSECtin treatment, STAT1 knockdown or overexpression and the distance between the edges of the scratch was quantified using ImageJ software. **F** The effect of LSECtin on STAT1 knockdown or overexpression GC cells migration were examined with Transwell migration assays, and the number of migrated cells was counted by ImageJ software. **G** The effect of LSECtin on STAT1 knockdown or overexpression GC cells migration were examined with EdU experiments. **H** Adhesion between GC cells and frozen mouse lymph node sections was detected in vitro by H&E staining (left). The right panel shows the cell number. Error bars indicate standard deviation (*n* = 3). Data, the means ± SD.

Published literature has confirmed the contribution of LSECtin members DC-SIGN and DC-SIGNR to GC carcinogenesis through upregulation of STAT family molecules [9, 10]. We inferred that LSECtin can also regulate STAT family members STAT1, STAT3, and STAT5A, which have been reported to be closely related with cancer [19]. Western blot results illustrated the downregulation of STAT1 expression after LSECtin treatment, whereas there were no significant differences in STAT3 or STAT5A expression (Fig. S2A). Based on Western blot and qPCR assays, LSECtin regulated STAT1 in a dose-dependent manner, and the alterations in STAT1 expression were distinguished after 24 hours of treatment (Fig. 3A, S2B). Given the important role of STAT1 activation in tumor metastasis, we then investigated whether LSECtin could modulate STAT1 phosphorylation. We found that LSECtin induced a reduction in the expression of phosphorylated STAT1 and STAT1, suggesting that the reduced phosphorylation may be due to a reduction in total protein expression (Fig. 3B-C). Accordingly, we focused on the potential role of STAT1 in gastric carcinogenesis, and we assessed the relationships among STAT1 expression, clinicopathological parameters, and prognosis as reported in TCGA and Kaplan-Meier Plotter databases. The results suggested increased STAT1 expression in GC tissue, but the downregulation of STAT1 expression was associated with distant metastasis and poor prognosis (Fig. 3D). The results of functional experiments showed that the reduction in STAT1 expression promoted the migration, proliferation, and adhesion of GC cells. Conversely, STAT1 overexpression resulted in a significant decrease in cell migration, proliferation, and adhesion (Fig. S2C-H). To determine the involvement of LSECtin-regulated STAT1 in GC tumorigenesis, we performed 5-ethynyl-2′-deoxyuridine (EdU), wound healing, and Transwell assays. Our studies suggested that LSECtin promoted the migration and proliferation of GC cells by knocking down STAT1 in vitro. The overexpression of STAT1 inhibited the migration and proliferation of GC cells caused by LSECtin treatment (Fig. 3E-G, S3A-C). Furthermore, LSECtin enhanced GC cell migration and proliferation after treatment with the STAT1 activation inhibitor fludarabine (Fig. S3D-H). In an in vitro adhesion assay, STAT1 knockdown enhanced the adhesion and restored closure induced by LSECtin-blocking antibody treatment, and an anti-LSECtin antibody was more likely to inhibit the adhesion of GC cells to the lymphoid sinus in STAT1-overexpressing GC cells (Fig. 3H). These pieces of evidences implied the pivotal role for STAT1 in LSECtin-induced tumorigenesis and lymphatic metastasis of GC cells.

### The circFBXL4/miR-146a-5p/STAT1 axis constructed in GC models

**Fig. 4:**
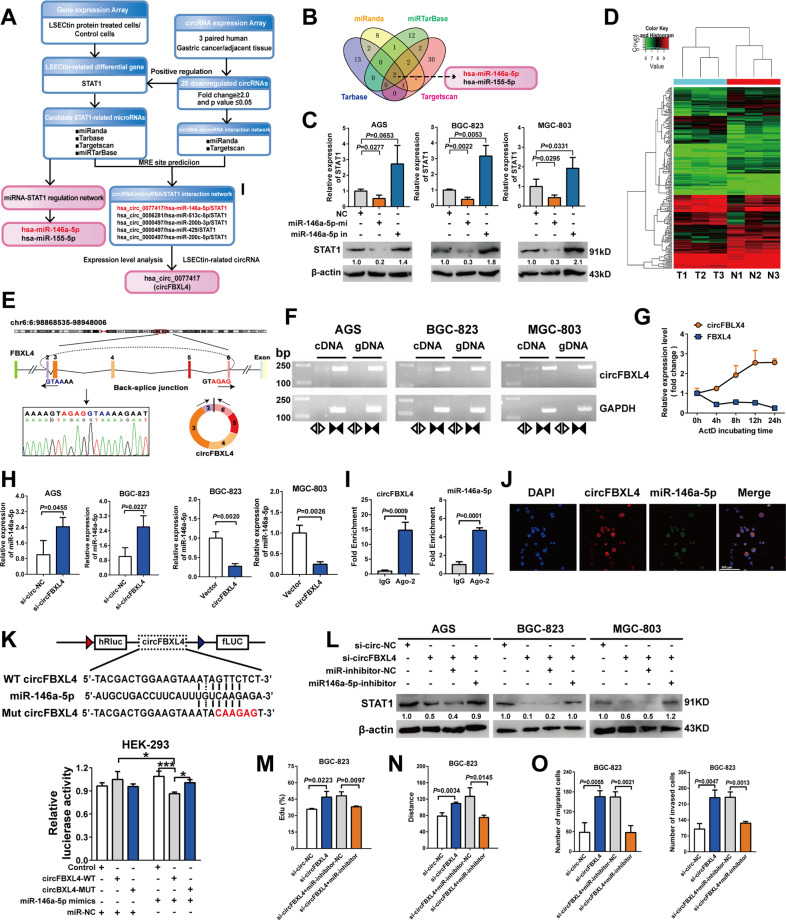
CircFBXL4/miR-146a-5p/STAT1 axis constructed in GC. **A** Flow chart of LSECtin-regulated STAT1-associated ceRNA network construction. **B** Venn diagram of miRNAs that may correlate with STAT1, as identified in four databases. **C** QPCR (top) and Western blot analysis (bottom) measures of STAT1 expression following transfection with miR-146a-5p mimics, a miR-146a-5p inhibitor or miR-NC. **D** Hierarchical clustering revealed the circRNA expression profile in GC tissues and adjusted normal tissues (greater than 1.5 fold difference in expression; *P* < 0.05). **E** Circulation site and formation process of circFBXL4. CircFBXL4 derived from FBXL4 exons 2–6 was 1507 nt in length. **F** Divergent primers (⊲⊳) spanning the spliced junction were used to amplify circFBXL4, and convergent primers (▶◀) were used to amplify FBXL4 mRNA, and agarose gel electrophoresis was used to identify circFBXL4 expression products in cDNA and genomic DNA. **G** qRT-PCR analysis of circFBXL4 and FBXL4 mRNA after treatment with actinomycin D at the indicated time points. **H** qPCR assays showing that silencing or overexpression circFBXL4 decreased or increased steady level of miR-146a-5p. **I** RIP followed by RT-qPCR was used to detect circFBXL4 and miR-146a-5p endogenously associated with Ago2 in BGC-823 cells. **J** FISH analysis of GC cells showing that circFBXL4 was colocalized with miR-146a-5p in cytoplasm, and nuclei were stained with DAPI. **K** Dual-luciferase activity was measured for the targeted binding sites in circFBXL4 and miR-146a-5p in HEK293 cells. **L** Western blot experiments showed that circFBXL4 and miR-146a-5p coregulate STAT1 expression in GC cells. **M** EdU assays indicated that miR-146a-5p inhibitors abolished the promotion of proliferation induced by circFBXL4 knockdown. **N** Wound healing assays showed that miR-146a-5p inhibitors suppressed the migration induced by circFBXL4 knockdown. **O** Transwell assays showed that miR-146a-5p inhibitors suppressed the migration and invasion induced by circFBXL4 knockdown. Error bars indicate standard deviation (*n* = 3). Data, the means ± SD.

DC-SIGN family of C-type lectins has always been closely related to non-coding RNA [9, 20]. In our experiment, we sought to determine whether circRNAs and miRNAs are involved in gastric LSECtin-mediated tumorigenesis using a preliminarily established regulatory axis (Fig. 4A). Four independent databases were used to predict the potential miRNAs involved the regulation of STAT1 expression. The concurrence of miRNA, miR-146a-5p and miR-155-5p was found in the four databases (Fig. 4B). A litereature review confirmed that miR-146a-5p directly targeted the on 3′- untranslated region (UTR) of STAT1 in a Dual-luciferase Reporter assay [21, 22]. The miR-146a-5p-binding sites were located in multibranching loops of STAT1 mRNA as shown in Fig. S4A. Additionally, miR-146a-5p expression was increased in GC tissue (Fig. S4B). MiR-146a-5p decreased STAT1 expression, and an miR-146a-5p inhibitor increased STAT1 expression in GC cells, as determined by qPCR and Western blot analysis (Fig. 4C, S4C). Our research demonstrated that miR-146a-5p negatively regulates STAT1 expression. In vitro assays all indicated that miR-146a-5p promote the progression of GC (Fig. S4D-F). Based on these findings, the involvement of miR-146a-5p/STAT1 axis in GC was confirmed.

A circRNA array was assessed to identify the differentially expressed circRNAs in 3 paired GC specimens (Fig. 4D, S5A–C, Table S5). To verify the proposed model, the expression of partial circRNAs from normal gastric mucosa cells and GC cell lines was measured (Fig. S5D). The circular feature of hsa_circ_0077417 (circFBXL4) was also identified in GC cells (Fig. 4E). Agarose gel electrophoresis results revealed that circFBXL4 was amplified only with divergent primers and cDNA, and no amplification products were observed with genomic DNA (gDNA), excluding those possibly formed through genome rearrangement (Fig. 4F). The linear transcripts of FBXL4 were identified with both random hexamer and oligo (dT)_18_ primers. However, circFBXL4 was nearly undetectable when oligo (dT)_18_ primers were used, consistent with its characteristic structure (Fig. S5E). Actinomycin D was used to inhibit transcription, and the expression of circFBXL4 and mFBXL4 was measured at different periods in BGC-823 cells. The results of actinomycin D showed that the half-life of circFBXL4 was much longer than that of the linear FBXL4 transcript, circFBXL4 was more stable than mFBXL4 (Fig. 4G). Cell Counting Kit-8 (CCK-8), Transwell and wound healing experiments further demonstrated that silencing circFBXL4 significantly promoted the proliferation and migration of GC cells (Fig. S5H–L).

To validate the existence of the circFBXL4/miR-146a-5p/STAT1 regulatory axis in GC, we verified the link between circFBXL4 and miR-146a-5p. CircFBXL4 knockdown or overexpression upregulated or downregulated miR-146a-5p expression were revealed by qPCR (Fig. 4H, S5M). The amount of circFBXL4 and miR-146a-5p pulled down with anti-Ago2 antibodies was significantly increased in BGC-823 cells compared to the amount pulled down with IgG. Ago2 can bind both circFBXL4 and miR-146a-5p, suggesting that circFBXL4 may bing to miR-146a-5p (Fig. 4I). FISH also revealed the colocalization of circFBXL4 and miR-146a-5p in the cytoplasm (Fig. 4J). The Luciferase reporter results showed that miR-146a-5p mimics only decreased the luciferase activity of the wild-type plasmid (Fig. 4K). These results suggested that circFBXL4 bound to miR-146a-5p. A Western blot analysis showed that circFBXL4 knockdown significantly inhibited STAT1 expression, while a miR-146a-5p inhibitor counteracted the repression as expected (Fig. 4L). Knockdown of circFBXL4 promoted the proliferation, migration and invasion in GC cell lines, and miR-146a-5p inhibitors reversed the promotion induced by circFBXL4 silencing (Fig. 4M-O, S5N-P). Based on the results above, we concluded that circFBXL4 inhibited tumorigenesis by acting as a competitive endogenous RNA (ceRNA) that induces miR-146a-5p-regulated STAT1 expression.

### LSECtin upregulates FN1 and CHD4 expression through STAT1 transcriptional regulation

**Fig. 5:**
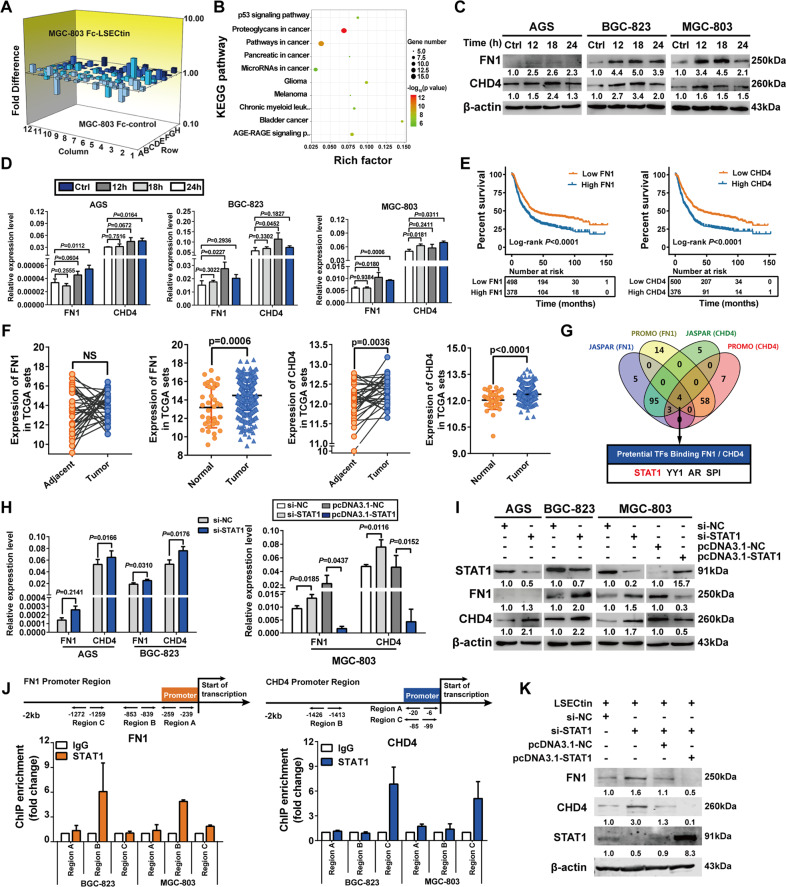
LSECtin induced FN1 and CHD4 expression through STAT1 transcriptional regulation. **A** The 3D profile according to the fold-change values of differentially expressed genes. **B** KEGG pathway analysis of common terms revealed enrichment of genes related to various events. **C**, **D** Differential expression of FN1 and CHD4 in GC cells treated with 1 μg/ml LSECtin protein (12 h, 18 h, and 24 h) were detected by Western blot (**C**) and qPCR (**D**), with controls treated with isotype IgG for 24 h. **E** Survival analysis of GC patients based on FN1 and CHD4 expression were analyzed by Kaplan-Meier plotter database. **F** FN1 and CHD4 expression levels in 415 samples of gasric cancer and 35 samples of normal tissue, FN1 and CHD4 expression levels in 32 tumor and matched normal samples from GC patients, **G** Venn diagram showing the transcription factors that coregulated FN1 and CHD4 identified in two databases. **H**, **I** The expression of FN1 and CHD4 in GC cells after STAT1 knockdown, or overexpression were analyzed by qPCR (**H**) and Western blot analysis (**I**). **J** Schematic representation of the STAT1-binding site in the FN1 and CHD4 promoters. Chromatin immunoprecipitation (ChIP) assays confirming FN1 and CHD4 promoter region enrichment with and anti-STAT1 antibody. **K** Western blot analysis of FN1 and CHD4 expression after the knockdown or overexpression of STAT1 in BGC-823 cells and LSECtin-induced. Error bars indicate standard deviation (*n* = 3). Data, the means ± SD.

The human gene expression array was used to detect differentially expressed molecules related to tumor metastasis between the LSECtin induction and the control group in MGC-803 cells, and a 3D profiles, scatter plots, and volcano plots were employed to visualize differences in gene expression (Fig. 5A, S6A–B). We next performed GO and KEGG pathway analyses and found that differentially expressed genes were involved in multiple pathways related to LSECtin-regulated tumorigenesis, including miRNAs in cancer (Fig. 5B, S6C). Among these genes, a difference was observed in FN1/CHD4 expression (Supplementary Table 4). The qRT-PCR and Western blot results revealed that LSECtin enhance FN1/CHD4 expression after 12 h (Fig. 5C–D). A number of studies have indicated the tumor-promoting properties of FN1 and CHD4 in various cancer cells [23–26], and these results were consistent with the GC data collected from the TCGA database. FN1 and CHD4 expression was significantly upregulated in GC tissues, and indicated poor survival (Fig. 5E–F). To understand the regulatory mechanisms between STAT1 and FN1/CHD4, the sequences of the FN1 and CHD4 promoter regions were analyzed using a promoter prediction database to identify coregulated transcription factor, including STAT1, YY1, AR and SPI (Fig. 5G). The qRT-PCR and Western blot results confirmed that STAT1 expression was negatively correlated with FN1/CHD4 expression (Fig. 5H–I, S6E). Indeed, STAT1 directly bound to the FN1 and CHD4 promoters, as shown by chromatin immunoprecipitation (Fig. 5J, S6F–G). The Western blot results showed that STAT1 was knocked down under the induction of LSECtin treatment, and the expression of FN1/CHD4 was increased (Fig. 5K). In conclusion, LSECin upregulated FN1/CHD4 expression by downregulating STAT1, which promoted GC progression.

### LSECtin upregulates FN1/CHD4 expression and promotes GC progression via the circFBXL4/miR-146a-5p/STAT1 axis

**Fig. 6:**
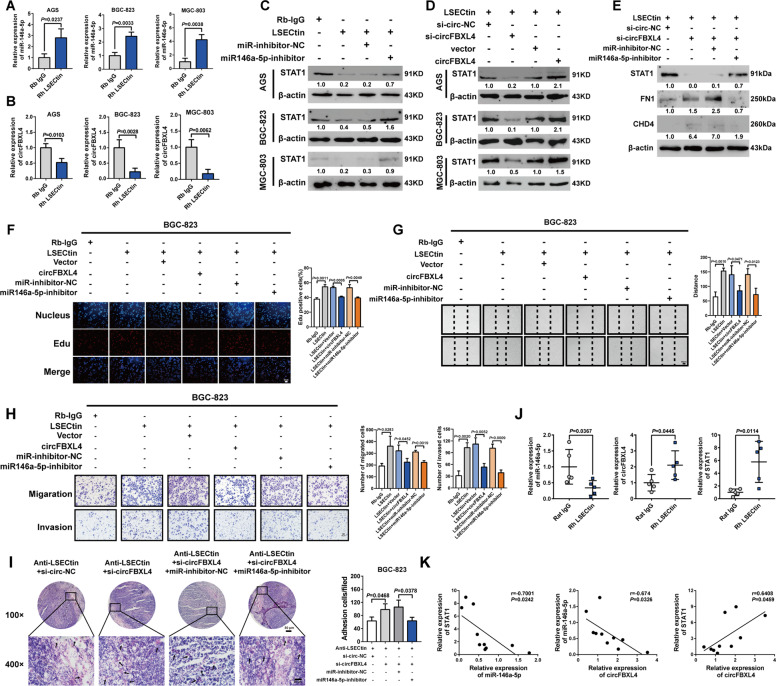
LSECtin upregulates FN1/CHD4 expression and promotes GC progression via the circFBXL4/miR-146a-5p/STAT1 axis. **A** LSECtin promoted the expression of miR-146a-5p in GC cells. **B** LSECtin inhibited the expression of hsa_circ_0077417 in GC cells. **C** Western blot experiments showed that LSECtin and miR-146a-5p coregulate STAT1 expression in GC cells. **D** Western blot experiments showed that LSECtin and circFBXL4 coregulate STAT1 expression in GC cells. **E** Western blot experiments showed that LSECtin upregulate FN1/CHD4 expression via the circFBXL4/miR-146a-5p axis. **F** Cell viability in different groups was detected by EdU assays, and the statistical analysis of EdU staining. **G** The migration of BGC-823 cells in different groups was detected by wound healing assays. **H** Transwell migration and invasion assays were performed to analyze the migration and invasion abilities of GC cells. **I** Adhesion between GC cells and frozen mouse lymph node sections in vitro was detected by H&E staining (left), The right panel shows the number of analysis of cells. **J** LSECtin expressed in lymph nodes of nude mice affected the expression of circFBXL4, miR-146a-5p and STAT1 in gastric cancer cells. **K** The expression of circFBXL4, miR-146a-5p and STAT1 in lymph nodes was correlated with statistical significance. Error bars indicate standard deviation (*n* = 3). Data, the means ± SD.

**Fig. 7:**
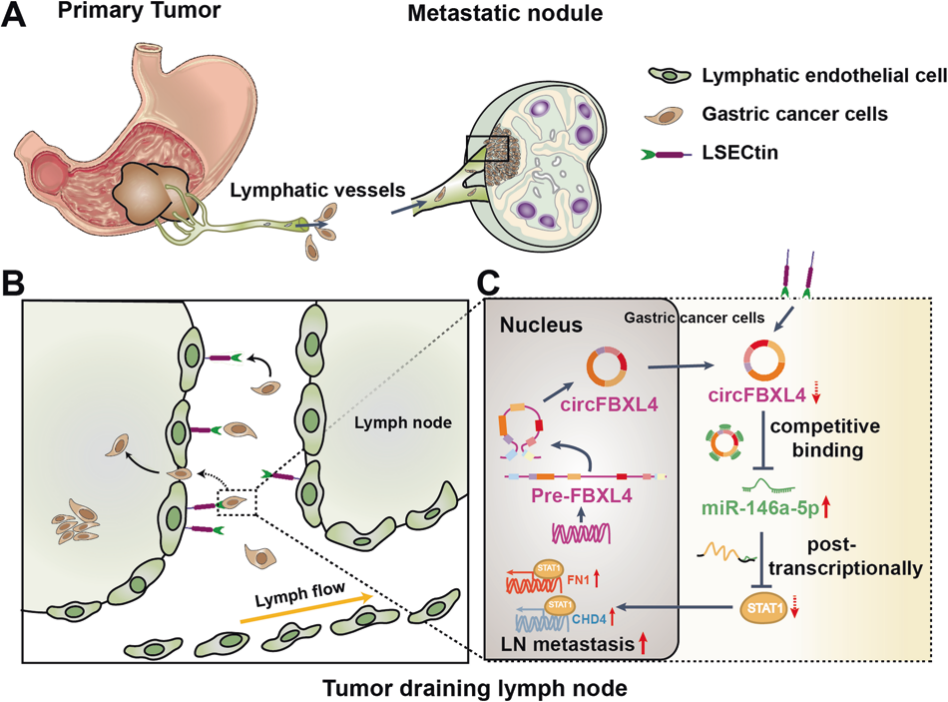
Schematic representation of the roles of LSECtin in GC progression. **A** GC cells infiltrate the surrounding tissues and lymphatic vessels, and reach the lymph nodes during lymphatic system metastases. **B** Under the action of molecules such as LSECtin, these cells adhere to the medullary sinus and subcapsular sinus, GC cells colonize to lymph nodes. **C** circFBXL4 can act through a ceRNA mechanism to “adsorb” miR-146a-5p to indirectly regulate the expression of STAT1, STAT1 affect the expression of FN1 and CHD4 as the transcription factor. LSECtin regulated STAT1, FN1 and CHD4 expression through the circFBXL4/miR-146a-5p axis, and affected the adhesion, proliferation, migration and invasion of GC cells, which might regulate GC lymphatic metastasis.

The qRT-PCR results showed that LSECtin upregulated the expression of miR-146a-5p and downregulated circFBXL4 expression (Fig. 6A-B). We hypothesized that LSECtin functions by regulating circFBXL4/miR-146a-5p axis. Western blot analysis confirmed that LSECtin-induced downregulation of STAT1 expression in GC cells was restored upon re-expression of a miR-146a-5p inhibitor and circFBXL4 (Fig. 6C-D). LSECtin decreased the expression of STAT1 and simultaneously increased FN1/CHD4 expression in circFBXL4 knockdown BGC-823 cells; importantly, these effects were abolished by miR-146a-5p inhibitors (Fig. 6E). EdU and Transwell experiments were performed for validation, and LSECtin-induced promotion of the proliferation, migration and invasion of GC cells were largely attenuated by the overexpression of circFBXL4 or the silencing of miR-146a-5p (Fig. 6F-H). The results of in vitro adhesion experiments showed that LSECtin expressed on lymph nodes affected the adhesion of GC cells to lymph nodes through the circFBXL4/miR-146a-5p axis (Fig. 6I). We used real-time quantitative PCR to detect the expression of circFBXL4, miR-146a-5p and STAT1 in the popliteal lymph node tissue of nude mice. The results showed that the expression of circFBXL4 and miR-146a-5p in the lymph node tissue of the nude mice was negatively correlated, miR-146a-5p negatively regulated the expression of STAT1, and the expression of circFBXL4, miR-146a-5p and STAT1 was correlated in vivo (Fig. 6J-K). These results suggest that circFBXL4 can act through a ceRNA mechanism to “adsorb” miR-146a-5p in vivo to indirectly regulate the expression of STAT1. LSECtin expressed in lymph nodes in nude mice affected the expression of circFBXL4, miR-146a-5p and STAT1 in GC cells, suggesting that LSECtin further promotes the development of gastric cancer by regulating the circFBXL4/miR-146a-5p/STAT1 axis. Taken together, LSECtin promoted the malignant and lymphatic metastasis of GC cells potentially through upregulating FN1/CHD4 expression by modulating the circFBXL4/miR-146a-5p/STAT1 axis (Fig. 7).

## Discussion

Many c-type lectins have been reported to be contributors of gastrointestinal tumor metastasis [7–9]. A study using transgenic mice expressing E-selectin only in the liver, showed that lung metastases caused by melanoma in normal mice formed liver metastases in transgenic mice. This finding indicated that E-selectin can mediate tumor cell metastasis to specific organs [27]. Another study showed that LSECtin expressed on the surface of liver sinusoidal endothelial cells is involved in colon cancer liver metastasis [8]. These data indicate that LSECtin is likely to be an important molecule that mediates metastasis. In this article, we confirmed the involvement of LSECtin in GC metastasis by discovering a previously unknown regulatory mechanism. We present a mechanistic diagram to illustrate the regulatory mechanism of LSECtin in GC (Fig. 7). In this study, the in vitro experimental results showed that LSECtin expressed on the surface of LSECs is a molecule promoting adhesion of GC cells, thereby promoting their migration and invasion and participating in the GC lymphatic metastasis. In the in vivo experiments, we incubated GC cells with LSECtin protein and then inoculated nude mice through their foot pads to block the adhesion of GC cells to LSECtin in lymph nodes, similar to procedures performed by others; the treatment blocked the metastasis of GC cells through lymph vessels. We speculated that GC cells separate from their original site, infiltrate the surrounding tissues and lymphatic vessels, and reach the lymph nodes during lymphatic system metastases. Under the action of molecules such as LSECtin, these cells adhere to the medullary sinus and subcapsular sinus. Additionally, GC cells colonize to lymph nodes. The increased level of LSECtin on endothelial cells further enhanced the invasion and metastasis ability of tumor cells. The extracellular fragments of the LSECtin protein fall off in the serum, resulting in a significant increase in expression levels.

We confirmed that LSECtin downregulates the expression of STAT1 in GC cells in a dose-dependent manner; and upregulates the expression of FN1/CHD4 through STAT1. However, the role of STAT1 in GC is controversial. It has been reported that kpnβ1 interacts with STAT1 and inhibits apoptosis through STAT1 nuclear import. However, many previous studies have confirmed that STAT1 is a tumor suppressor gene that is related to the formation and metastasis of many tumors and plays an important role in regulating tumor cell proliferation, survival, and angiogenesis [28]. The IFNγ/STAT1 signaling pathway is involved in MTMR2-enhanced invasion and metastasis of GC cells [29]. In GC, STAT1 expression showed negative correlations with the depth of wall invasion, which may be a indicator of clinical outcome [30]. In this study, the results of database survival analysis confirmed that GC patients with higher expression of STAT1 experience better survival outcomes. Therefore, we clarified the participation and inhibitory function of STAT1 in the development of GC; however, other potential regulatory axes cannot be excluded, like the activation mechanism of STAT1 through phosphorylation [25, 27].

The abnormally expressed circRNAs in GC inhibited the expression of targeted genes by functioning as miRNA sponge [31, 32]. To better understand the circRNA/miRNA regulatory mechanism of LSECtin in GC, we performed database site prediction assays with differing methods to establish a screening procedure for related STAT1 regulatory axes. According to bioinformatic analysis using our chip array, we hypothesized that miR-146a-5p binds to circFBXL4. Some evidence suggested that miR-146a-5p exhibits pro-oncogenic properties in GC. Patients with high miR-146a-5p levels were found to be more likely to have an advanced tumor stage and lymph node metastasis [33, 34]. Consistent with these findings, we found that overexpression of miR-146a-5p promoted oncogenesis by inhibiting STAT1 expression. By performing rescue experiments, we demonstrated that miR-146a-5p counteracts cancer-promoting effects by reducing the expression of circFBXL4 in GC cells, representing a novel regulatory relationship between circFBXL4 and miR-146a-5p. We validated other predicted regulatory axes, as shown in Fig. 4A; however, hsa_circ_0000497 could not be quantified due to the low expression of valid indicators (data not shown). The qPCR results showed that LSECtin caused circFBXL4 downregulation but had no effect on hsa_circ_0056281. These results suggested that LSECtin specifically downregulated the expression of circFBXL4, which is a sponge of miR-146a-5p.

Thus, LSECtin regulated STAT1, FN1 and CHD4 expression through the circFBXL4/miR-146a-5p axis, which was confirmed by Western blot analysis, and affected the adhesion, proliferation, migration and invasion of GC cells through the circFBXL4/miR-146a-5p/STAT1 axis, which might regulate GC lymphatic metastasis. However, the mechanism by which LSECtin downregulated circFBXL4 is not clear. Considering these findings, we predicted the transcription factor of the parent gene FBXL4 of circFBXL4 through databases. STAT1 was likely to affect the production of circFBXL4 as the transcription factor of the parent gene FBXL4. We speculated that LSECtin affects the transcription level of FBXL4 through the transcriptional regulation of STAT1, thereby indirectly affecting the expression of circFBXL4. The role of LSECtin in regulation of GC progression appears more complex and is not limited to the mechanism uncovered by the present study, and further studies are required to clarify the complex regulatory network.

## Supplementary information

Supplementary information.

Language editing certificate

aj-checklist

cddis-author-contribution-form

Original Data File

## Data Availability

The data supporting our findings can be found in the supplementary data.
