# Supplementary material for: Novel roles of LSECtin in gastric cancer cell adhesion, migration, invasion, and lymphatic metastasis
Source: Cell Death Dis. 2022 Jul 11;13(7):593. doi: 10.1038/s41419-022-05026-x (PMC9276708; doi:10.1038/s41419-022-05026-x)
Supplement: Supplementary file 1 — Supplementary information. [file 41419_2022_5026_MOESM1_ESM.pdf]

Supplementary information

Supplementary file 1：Supplemental Materials and Methods

Genomic DNA and RNA extraction

Genomic DNA (gDNA) was extracted from cells using an Easypure™ Genomic DNA kit (TransGen Biotech, Beijing, China). Total RNA was extracted from cultured cells using TRIZOL reagent (TaKaRa, Tokyo, Japan), row RNA concentration was measured by NanoDrop 2000 (Thermo Fisher Scientific, Waltham, MA, USA)

Quantitative real-time PCR (qPCR)

RNA was reverse transcribed into cDNA for circRNA, mRNA or miRNA by using random (TaKaRa, Japan), oligo(dT)18 primers (Thermo Fisher Scientific) or stem-loop primers, respectively. Primers are listed in Supplementary file Table S1. q-PCR analyses were performed with SuperReal PreMix Plus (TIANGEN) or miRNA Universal SYBR qPCR Master Mix (vazyme, Nanjing, China) on the Thermal Cycler Dice Real-Time System (TaKaRa). GAPDH or U6 was used as the internal reference, the 2-ΔΔCt method was adopted to calculate the relative expression of target genes.

Western blot analysis

The protein was extracted from cultured cells using a total protein extraction kit (KeyGen, Jiangsu, China) and the protein concentration was measured via BCA Protein Assay Kit (Beyotime, China) or (Sevenbio, Beijing, China). Protein was electrophoresed in 8% SDS polyacrylamide gels, transferred onto 0.22 μm nitrocellulose membranes (Pall Corporation, New York, USA). The membranes were then incubated with assigned primary antibodies and

corresponding secondary antibodies. Signals were detected with electrochemical luminescence reagent (Advansta, USA). β-actin (1:500, ZSGB-BIO) was used as loading control, anti-STAT5A (1:500) was purchased from Elabscience (Wuhan, China), anti-STAT1 (1:500), Anti-STAT3 (1:10000) were purchased from BD Biosciences. Anti-FN1(1:1500) was purchased from Proteintech (Wuhan, China), Anti-CHD4(1:10000) was purchased from Abcam (Cambridge, MA, USA). Anti-rabbit IgG (1:2000, ZSGB-BIO) and anti-mouse IgG (1:2000, ZSGB-BIO) HRP-conjugated secondary antibodies were used.

Colony formation assay

GC cells with the recombinant LSECtin protein or with control IgG were seeded 1000 cells per well in six-well plates. All cell lines were cultured for 2 weeks, colonies were stained by 0.5% crystal violet and counted under optical microscope (Olympus IX71, Tokyo, Japan).

Wound healing assay

3×10^5 cells were seeded in 12-well plates and incubated overnight. A vertical scratch was produced using a 10 μL pipette tip, then the plate was washed twice, and cultured in the serum-free medium. The scratch width as a migration index was observed and photographed under a microscope (Olympus) at 0 and 24 hours, measured by ImageJ software.

CCK8 assay and 5-Ethynyl-2-deoxyuridine (EdU) assay

The proliferation of cells was tested by the CCK8 assay following Cell Counting Kit 8 (MCE, Monmouth Junction, NJ, USA) manufacturer's instructions. Enzyme-labeling instrument was

used for the measurement of OD value at 450 nm wavelength at day 1, 2, 3, and 4. EdU assay kit (Beyotime) was applied to detect DNA synthesis, which could also reflect cell proliferation. hoechst 33342 was added for nuclear staining. EdU cells were photographed and counted under Olympus fluorescence microscopy.

Transwell assay

The transwell assay (24-well, Corning, NY, USA) was used to measure the cell migration and invasion. Briefly, 3×10^5 or 4.0×10^5 cells with 200 ul serum-free medium were added to the upper chamber of migratory or invasion assay. The chamber pre-coated with Matrigel (BD Biosciences) was used for invasion assay. Cells were incubated for 24 h, then fixed, and next stained with 0.5% Crystal Violet Solution. The average value of cell counting was calculated from five different microscopic fields.

Supplementary file 2: Table S1. Primers and probes used in the study.

Primer sequences for RT-PCR and qPCR

Company	Gene	primer sequences
Takara	LSECtin	F: 5′-GCCATGGACACCACCAGGTACAGC-3′
		R: 5′-GACTCAGCAGTTGTGCCTTTTCTC-3′
	β-actin	F:5′-ATCTGGCACCAAACACCTTCTACAATGAGCTGCG -3′
		R:5′-CGTCATACTCCTGCTTGCTGATCCACATCTGC -3′
genepharma	hsa_circ_0097100	F: 5′-CCAAACATCCAGCCACAAGA-3′
		R: 5′-ACCCACTACTGTTGCCTGAA-3′
	hsa_circ_0073244	F: 5′-TCACTGCCTCCAGCATCTT-3′
		R: 5′-TGTCTTCATTGGTGCCTTTC-3′
	hsa_circ_0027966	F: 5′-AGTCTGCTCAAGTTCTACTGCT-3′
		R: 5′-TCAGAACCGCACAAACACTG-3′
	hsa_circ_0056281	F: 5′-CACCTGCCGACAGTGGTT-3′
		R: 5′-CATTCAGTGCTTGGGATGTTT-3′
	hsa_circ_0077417	F: 5′-GCCTTGAATTGTCTTGCAGCCAC-3′
	(circFBXL4)	R: 5′-ATGCTGACATGTGACATTCTTTTACCTCT-3′
sangon	STAT1	F: 5'-TGGATCAGCTGCAGAACTGG-3'
		R: 5'-GAAGGTGCGGTCCCATAACA-3'
	STAT3	F: 5′-GGAGCATCCTGAAGCTGACC-3′
		R: 5′-GTAGGCGCCTCAGTCGTATC-3′
	STAT5A	F: 5′-ACCTGTGGTTGTCATCGTCC-3′
		R: 5′-TTTGTCAGGCACGGCAAATG-3′
	FN1	F: 5′-GGTGACACTTATGAGCGTCCTAAA-3′
		R: 5′-AACATGTAACCACCAGTCTCATGTG-3′
	CHD4	F: 5′-ACATTGCCAAGGAGGACC-3′
		R: 5′-CACTTCTGCATAGGGCTCAG-3′
	YY1	F: 5′-AAGAAGTGGGAGCAGAAGCA-3′
		R: 5′-CAACCACTGCTCATGGCAA-3′
	AR	F: 5′- GGTTACACCAAAGGGCTAGAA -3′
		R: 5′- GACTTGTAGAGAGACAGGGTAGA -3′
	SP1	F: 5′- CCACCATGAGCGACCAAGAT -3′
		R: 5′- AAGGCACCACCACCATTACC -3′
	FBXL4	F: 5′-CTTATGGGACATGGTGGGATCAG-3′
		R: 5′-AGCTGGTGGATTTGGGGAATAAG-3′
	Divergent GAPDH-F	F: 5′-GGCCTCCAAGGAGTAAGA-3′
		R: 5′-GCCCAATACGACCAAATCA-3′
	GAPDH	F: 5′-CCTCAAGATCATCAGCAAT-3′

		R: 5′-CCATCCACAGTCTTCTGGGT-3′
	hsa-miR-146a-5p (Reverse Transcription)	GTCGTATCCAGTGCAGGGTCCGAGGTATTCGCACTGGATACGACAACCCA
	hsa-miR-146a-5p	F: 5′-CGCGTGAGAACTGAATTCCA-3′
	U6 (Reverse Transcription)	AACGCTTCACGAATTTGCGT
	U6	F: 5′-CTCGCTTCGGCAGCACA-3′<br>R: 5′-AACGCTTCACGAATTTGCGT

Primer sequences for CHIP assay

Company	Gene	primer sequences
sangon	FN1 Region A	F: 5′- GGATTTCCAGGTATCCTTCCCTC-3′<br>R: 5′- TGACCCACACTCCAAATTGAAAGA-3′
	FN1 Region B	F: 5′- AGGACTGATGTGATGTGTTGCT-3′<br>R: 5′- ATTACAGAATGCCCCCTCCATC-3′
	FN1 Region C	F: 5′- GGTGAACGTGGGAAAGGACA-3′<br>R: 5′- GGGAGCAAAGGGGAACAGAG-3′
	CHD4 Region A	F: 5′- GTGGAGTCTCTTAAGAAGGAAGGACA-3′<br>R: 5′- CGTGGTCCTTCTAGAGGCTGG-3′
	CHD4 Region B	F: 5′- GCCGAGTGTCCGTTGTAGT-3′<br>R: 5′- ATAATAAGCACCCCTCGCAGCA-3′
	CHD4 Region C	F: 5′- AGGGAACAGATAGGAGATCATGT-3′<br>R: 5′- TGCCAAGGGAGAACTGCT-3′

Probe sequences for FISH assay

Company	Gene	primer sequences
genepharma	hsa_circ_0077417	5′- CY3-CTGACATGTGACATTCTTTTACCTCTACTTTTGTTCG -3′
	hsa-miR-146a-5p	5′- FAM-AACCCATGGAATTCAGTTCTCA -3′

Supplementary file 3: Table S2. Target sequences for knockdown and overexpression.

Company	Gene	sequences
genepharma	overexpression LSECin, transcript variant 8	NM_198492.3
	Overexpression STAT1, transcript variant α	NM_007315.4
	si-hsa_circ_0077417 (circFBXL4)	5′- CAAAAGUAGAGGUAAAAGATT<br>UCUUUUACCUCUACUUUUGTT-3′
	si-STAT1	sense: 5′- CUCAUUCCGUGGACGAGGUdTdT -3′<br>antisense: 5′- ACCUCGUCCACGGAAUGAGdTdT -3′
	si-NC-	sense: 5′- UUCUCCGAACGUGUCACGUdTdT -3′<br>antisense: 5′- ACGUGACACGUUCGGAGAAdTdT -3′
	miR-146a-5p mimics	sense: 5′- UGAGAACUGAAUUCCAUGGGUU -3′<br>antisense: 5′- CCCAUGGAAUUCAGUUCUCAUU -3′
	miR-146a-5p inhibitor	5′-AACCCAUGGAAUUCAGUUCUCA-3′
	miRNA-NC	sense: 5′- UUCUCCGAACGUGUCACGUdTdT -3′<br>antisense: 5′- ACGUGACACGUUCGGAGAAdTdT -3′

Supplementary file 4: Table S3. Databases used in this study.

Dataset	Source
Genotype-Tissue Expression (GTEx)	https://www.gtexportal.org/
The Cancer Genome Atlas (TCGA)	https://portal.gdc.cancer.gov
The Human Protein Atlas	https://www.proteinatlas.org/
GDAC Firehose	http://gdac.broadinstitute.org/
KM Plotter	http://kmplot.com/analysis
OmicsBean	http://www.omicsbean.cn/account_center/
UCSC Genome Browser	http://genome.ucsc.edu/
PROMO	http://alggen.lsi.upc.es/
JASPAR	http://jaspar.genereg.net/
miRanda	http://www.microrna.org
TargetScan	http://www.targetscan.org/
Tarbase	http://microrna.gr/tarbase/
miRTarBase	http://mirtarbase.mbc.nctu.edu.tw/
Vienna RNA Web Services	http://rna.tbi.univie.ac.at/

Supplementary file 5: Table S4. Positive and negative transcriptional regulation by LSECin-reduced in MGC-803 cells

Symbol	RefSeq	Description	p value	Fold Regulation	Comments
APC	NM_000038	Adenomatous polyposis coli	0.438053	1.437304	B
BRMS1	NM_015399	Breast cancer metastasis suppressor 1	0.39819	-1.3724	OKAY
CCL7	NM_006273	Chemokine (C-C motif) ligand 7	0.941716	-1.11272	C
CD44	NM_000610	CD44 molecule (Indian blood group)	0.257056	1.150436	OKAY
CD82	NM_002231	CD82 molecule	0.303211	-1.28644	OKAY
CDH1	NM_004360	Cadherin 1, type 1, E-cadherin (epithelial)	0.575858	1.134011	OKAY
CDH11	NM_001797	Cadherin 11, type 2, OB-cadherin (osteoblast)	0.941716	-1.11272	C
CDH6	NM_004932	Cadherin 6, type 2, K-cadherin (fetal kidney)	0.941716	-1.11272	C
CDKN2A	NM_000077	Cyclin-dependent kinase inhibitor 2A (melanoma, p16, inhibits CDK4)	0.444247	-1.11085	OKAY
CHD4	NM_001273	Chromodomain helicase DNA binding protein 4	0.006436	1.469298	OKAY
COL4A2	NM_001846	Collagen, type IV, alpha 2	0.767418	-1.05624	OKAY
CST7	NM_003650	Cystatin F (leukocystatin)	0.653741	1.432535	B
CTBP1	NM_001328	C-terminal binding protein 1	0.531739	-1.17941	OKAY
CTNNA1	NM_001903	Catenin (cadherin-associated protein), alpha 1, 102kDa	0.069541	1.314897	OKAY
CTSK	NM_000396	Cathepsin K	0.032663	-2.0814	OKAY
CTSL	NM_001912	Cathepsin L1	0.54156	-1.2385	OKAY
CXCL12	NM_000609	Chemokine (C-X-C motif) ligand 12	0.941716	-1.11272	C
CXCR2	NM_001557	Chemokine (C-X-C motif) receptor 2	0.709487	1.068989	OKAY
CXCR4	NM_003467	Chemokine (C-X-C motif) receptor 4	0.434009	1.124785	OKAY
DENR	NM_003677	Density-regulated protein	0.351481	1.18624	OKAY
EPHB2	NM_004442	EPH receptor B2	0.515991	-1.09995	OKAY
ETV4	NM_001986	Ets variant 4	0.02815	-2.49706	OKAY
EWSR1	NM_005243	Ewing sarcoma breakpoint region 1	0.752024	1.121713	OKAY

FAT1	NM_005245	FAT tumor suppressor homolog 1 (Drosophila)	0.419371	1.094797	OKAY
FGFR4	NM_002011	Fibroblast growth factor receptor 4	0.430231	1.702411	B
FLT4	NM_002020	Fms-related tyrosine kinase 4	0.294126	-1.28264	OKAY
FN1	NM_002026	Fibronectin 1	0.047732	1.231894	OKAY
FXYD5	NM_014164	FXYD domain containing ion transport regulator 5	0.473154	-1.12467	OKAY
GNRH1	NM_000825	Gonadotropin-releasing hormone 1 (luteinizing-releasing hormone)	0.437771	-2.20792	B
HGF	NM_000601	Hepatocyte growth factor (hepapoietin A; scatter factor)	0.941716	-1.11272	C
HPSE	NM_006665	Heparanase	0.83797	-1.08803	OKAY
HRAS	NM_005343	V-Ha-ras Harvey rat sarcoma viral oncogene homolog	0.301705	-1.322	OKAY
HTATIP2	NM_006410	HIV-1 Tat interactive protein 2, 30kDa	0.725035	-1.09985	OKAY
IGF1	NM_000618	Insulin-like growth factor 1 (somatomedin C)	0.784732	-1.21017	OKAY
IL18	NM_001562	Interleukin 18 (interferon-gamma-inducing factor)	0.548008	-1.15815	OKAY
IL1B	NM_000576	Interleukin 1, beta	0.442934	-1.61621	OKAY
ITGA7	NM_002206	Integrin, alpha 7	0.885548	1.111983	B
ITGB3	NM_000212	Integrin, beta 3 (platelet glycoprotein IIIa, antigen CD61)	0.500032	1.146744	OKAY
KISS1	NM_002256	KiSS-1 metastasis-suppressor	0.095299	2.341258	B
KISS1R	NM_032551	KISS1 receptor	0.941716	-1.11272	C
KRAS	NM_004985	V-Ki-ras2 Kirsten rat sarcoma viral oncogene homolog	0.5597	1.139427	OKAY
MCAM	NM_006500	Melanoma cell adhesion molecule	0.168785	-1.29697	OKAY
MDM2	NM_002392	Mdm2 p53 binding protein homolog (mouse)	0.416072	1.209495	OKAY
MET	NM_000245	Met proto-oncogene (hepatocyte growth factor receptor)	0.968884	-1.0691	OKAY
METAP2	NM_006838	Methionyl aminopeptidase 2	0.438876	1.367225	OKAY
MGAT5	NM_002410	Mannosyl (alpha-1,6-)-glycoprotein beta-1,6-N-acetyl-glucosaminyltransferase	0.45006	1.211376	B
MMP10	NM_002425	Matrix metallopeptidase 10 (stromelysin 2)	0.616525	1.063492	B
MMP11	NM_005940	Matrix metallopeptidase 11 (stromelysin 3)	0.633542	1.06558	OKAY

MMP13	NM_002427	Matrix metallopeptidase 13 (collagenase 3)	0.196956	1.496426	B
MMP2	NM_004530	Matrix metallopeptidase 2 (gelatinase A, 72kDa gelatinase, 72kDa type IV collagenase)	0.499929	-1.11727	OKAY
MMP3	NM_002422	Matrix metallopeptidase 3 (stromelysin 1, progelatinase)	0.241062	1.327134	B
MMP7	NM_002423	Matrix metallopeptidase 7 (matrilysin, uterine)	0.327655	1.648755	A
MMP9	NM_004994	Matrix metallopeptidase 9 (gelatinase B, 92kDa gelatinase, 92kDa type IV collagenase)	0.034077	-1.73309	OKAY
MTA1	NM_004689	Metastasis associated 1	0.997312	1.03201	OKAY
MTSS1	NM_014751	Metastasis suppressor 1	0.907747	-1.12187	B
MYC	NM_002467	V-myc myelocytomatosis viral oncogene homolog (avian)	0.989481	1.023254	OKAY
MYCL	NM_005376	V-myc myelocytomatosis viral oncogene homolog 1, lung carcinoma derived (avian)	0.828161	1.020831	B
NF2	NM_000268	Neurofibromin 2 (merlin)	0.673838	-1.11124	B
NME1	NM_000269	Non-metastatic cells 1, protein (NM23A) expressed in	0.256184	-1.15041	OKAY
NME4	NM_005009	Non-metastatic cells 4, protein expressed in	0.247389	-1.21404	OKAY
NR4A3	NM_006981	Nuclear receptor subfamily 4, group A, member 3	0.928218	-1.13791	OKAY
PLAUR	NM_002659	Plasminogen activator, urokinase receptor	0.061764	-1.37303	OKAY
PNN	NM_002687	Pinin, desmosome associated protein	0.353968	1.343566	OKAY
PTEN	NM_000314	Phosphatase and tensin homolog	0.445862	1.347252	OKAY
RB1	NM_000321	Retinoblastoma 1	0.441932	1.254058	OKAY
RORB	NM_006914	RAR-related orphan receptor B	0.587815	-1.11285	B
RPSA	NM_002295	Ribosomal protein SA	0.840943	-1.12381	OKAY
SERPINE1	NM_000602	Serpin peptidase inhibitor, clade E (nexin, plasminogen activator inhibitor type 1), member 1	0.36268	-1.23971	OKAY
SET	NM_003011	SET nuclear oncogene	0.160907	1.280094	OKAY
SMAD2	NM_005901	SMAD family member 2	0.888518	-1.09318	OKAY

SMAD4	NM_005359	SMAD family member 4	0.240414	1.440229	OKAY
SRC	NM_005417	V-src sarcoma (Schmidt-Ruppin A-2) viral oncogene homolog (avian)	0.616017	-1.37165	B
SSTR2	NM_001050	Somatostatin receptor 2	0.828306	-1.02753	B
SYK	NM_003177	Spleen tyrosine kinase	0.941716	-1.11272	C
TCF20	NM_005650	Transcription factor 20 (AR1)	0.639317	1.093421	OKAY
TGFB1	NM_000660	Transforming growth factor, beta 1	0.248561	-1.3087	OKAY
TIMP2	NM_003255	TIMP metallopeptidase inhibitor 2	0.136599	1.177798	OKAY
TIMP3	NM_000362	TIMP metallopeptidase inhibitor 3	0.25144	-1.21614	OKAY
TIMP4	NM_003256	TIMP metallopeptidase inhibitor 4	0.95758	-1.20065	OKAY
TNFSF10	NM_003810	Tumor necrosis factor (ligand) superfamily, member 10	0.486188	1.470209	A
TP53	NM_000546	Tumor protein p53	0.971027	-1.03106	OKAY
TRPM1	NM_002420	Transient receptor potential cation channel, subfamily M, member 1	0.941716	-1.11272	C
TSHR	NM_000369	Thyroid stimulating hormone receptor	0.941716	-1.11272	C
VEGFA	NM_003376	Vascular endothelial growth factor A	0.25337	-1.31891	OKAY
ACTB	NM_001101	Actin, beta	0.196933	1.245451	OKAY
B2M	NM_004048	Beta-2-microglobulin	0.873538	-1.22524	OKAY
GAPDH	NM_002046	Glyceraldehyde-3-phosphate dehydrogenase	0.89884	1.027091	OKAY
HPRT1	NM_000194	Hypoxanthine phosphoribosyltransferase 1	0.342481	1.209938	OKAY
RPLP0	NM_001002	Ribosomal protein, large, P0	0.141741	-1.26322	OKAY

A: This gene's average threshold cycle is relatively high (> 30) in either the control or the test sample, and is reasonably low in the other sample (< 30). These data mean that the gene's expression is relatively low in one sample and reasonably detected in the other sample suggesting that the actual fold-change value is at least as large as the calculated and reported fold-change result. This fold-change result may also have greater variations if p value > 0.05; therefore, it is important to have a sufficient number of biological replicates to validate the result for this gene.

B: This gene's average threshold cycle is relatively high (> 30), meaning that its relative expression level is low, in both control and test

samples, and the p-value for the fold-change is either unavailable or relatively high ($p > 0.05$). This fold-change result may also have greater variations; therefore, it is important to have a sufficient number of biological replicates to validate the result for this gene.

C: This gene's average threshold cycle is either not determined or greater than the defined cut-off (deault 35), in both samples meaning that its expression was undetected, making this fold-change result erroneous and un-interpretable.

Supplementary file 6: Table S5. Differentially expressed circRNAs of down regulaion in gastric cancer.

probeID	P-value	FC (abs)	Regulation	Alias	circRNA_type	miRNA Binding Sites				
ASCRP3009602	0.001102449	4.3109455	down	hsa_circ_0027966	exonic	hsa-miR-6501-5p	hsa-miR-17-3p	hsa-miR-653-3p	hsa-miR-29b-1-5p	hsa-miR-4506
ASCRP3001814	0.000675962	4.2836462	down	hsa_circ_0097100	exonic	hsa-miR-6501-5p	hsa-miR-3064-3p	hsa-miR-424-5p	hsa-miR-15b-5p	hsa-miR-6838-5p
ASCRP3009428	0.041487479	3.8312031	down	hsa_circ_0073244	exonic	hsa-miR-218-1-3p	hsa-miR-335-3p	hsa-miR-33a-3p	hsa-miR-589-3p	hsa-miR-134-5p
ASCRP3009292	0.009114575	3.1071768	down	hsa_circ_0056281	exonic	hsa-miR-892c-3p	hsa-miR-597-3p	hsa-miR-513c-5p	hsa-miR-181b-5p	hsa-miR-635
ASCRP3002173	0.002793197	3.0950285	down	hsa_circ_0000497	exonic	hsa-miR-200b-3p	hsa-miR-491-5p	hsa-miR-141-5p	hsa-miR-200c-3p	hsa-miR-429
ASCRP3007576	0.007111144	3.0873926	down	hsa_circ_0006528	exonic	hsa-miR-892a	hsa-miR-7-5p	hsa-miR-329-5p	hsa-miR-204-3p	hsa-miR-449c-5p
ASCRP3001896	0.031758793	2.8110134	down	hsa_circ_0001400	exonic	hsa-miR-425-5p	hsa-miR-485-3p	hsa-miR-637	hsa-miR-519d-3p	hsa-miR-29b-1-5p
ASCRP3012554	0.046853695	2.7764378	down	hsa_circ_0077736	exonic	hsa-miR-6507-3p	hsa-miR-6077	hsa-miR-8065	hsa-miR-1248	hsa-miR-942-5p
ASCRP3010196	0.010017895	2.7364418	down	hsa_circ_0007292	exonic	hsa-miR-103a-2-5p	hsa-miR-19b-2-5p	hsa-miR-335-3p	hsa-miR-153-5p	hsa-miR-376b-3p
ASCRP3008095	0.035879103	2.654156	down	hsa_circ_0002664	exonic	hsa-miR-374a-3p	hsa-miR-550a-5p	hsa-miR-550a-3-5p	hsa-miR-181a-5p	hsa-miR-101-5p
ASCRP3009067	0.009204668	2.6484644	down	hsa_circ_0008132	exonic	hsa-miR-4753-3p	hsa-miR-892a	hsa-miR-4693-5p	hsa-miR-7-5p	hsa-miR-6881-3p
ASCRP3005903	0.027506859	2.6062989	down	hsa_circ_0072309	exonic	hsa-miR-875-3p	hsa-miR-516b-5p	hsa-miR-525-5p	hsa-miR-515-5p	hsa-miR-520a-5p
ASCRP3000526	0.029889663	2.6058737	down	hsa_circ_0001678	exonic	hsa-miR-8060	hsa-miR-134-5p	hsa-miR-1294	hsa-miR-4709-3p	hsa-miR-3945
ASCRP3001374	0.010470134	2.5955736	down	hsa_circ_0005652	exonic	hsa-miR-329-5p	hsa-miR-671-5p	hsa-miR-515-5p	hsa-miR-20b-3p	hsa-miR-541-3p
ASCRP3002775	0.001281505	2.4415774	down	hsa_circ_0100928	exonic	hsa-miR-8089	hsa-miR-3150a-3p	hsa-miR-6881-3p	hsa-miR-4691-5p	hsa-miR-4646-5p
ASCRP3005290	0.002908037	2.4355504	down	hsa_circ_0007132	exonic	hsa-miR-638	hsa-miR-92a-2-5p	hsa-miR-542-5p	hsa-miR-769-3p	hsa-miR-661
ASCRP3009192	0.045056042	2.4093436	down	hsa_circ_0142447	exonic	hsa-miR-8060	hsa-miR-4658	hsa-miR-6790-5p	hsa-miR-134-5p	hsa-miR-1294
ASCRP3006741	0.017759737	2.3761981	down	hsa_circ_0000231	exonic	hsa-miR-135b-5p	hsa-miR-135a-5p	hsa-miR-518c-5p	hsa-miR-552-3p	hsa-miR-30c-1-3p
ASCRP3013398	0.034773329	2.3309026	down	hsa_circ_0009158	exonic	hsa-miR-22-5p	hsa-miR-598-3p	hsa-miR-3682-5p	hsa-miR-3199	hsa-miR-4677-5p
ASCRP3000708	0.037013934	2.3174977	down	hsa_circ_0008337	exonic	hsa-miR-22-5p	hsa-miR-3692-5p	hsa-miR-670-3p	hsa-miR-384	hsa-miR-598-3p
ASCRP3008281	0.046765709	2.2525026	down	hsa_circ_0006554	exonic	hsa-miR-15b-3p	hsa-miR-30d-3p	hsa-miR-30e-3p	hsa-miR-19b-3p	hsa-miR-301a-3p
ASCRP3000976	0.037354399	2.2071125	down	hsa_circ_0077417	exonic	hsa-miR-3692-3p	hsa-miR-149-5p	hsa-miR-146a-5p	hsa-miR-2110	hsa-miR-450a-2-3p
ASCRP3006776	0.014418646	2.1552922	down	hsa_circ_0017667	exonic	hsa-miR-6856-3p	hsa-miR-3160-5p	hsa-miR-5009-5p	hsa-miR-103a-2-5p	hsa-miR-4659b-3p
ASCRP3012746	0.008528096	2.1432898	down	hsa_circ_0069618	exonic	hsa-miR-620	hsa-miR-542-3p	hsa-miR-22-5p	hsa-miR-647	hsa-miR-570-3p
ASCRP3006721	0.04138492	2.1036666	down	hsa_circ_0058495	exonic	hsa-miR-103a-2-5p	hsa-miR-525-5p	hsa-miR-182-5p	hsa-miR-520a-5p	hsa-miR-22-5p
ASCRP3012555	0.014241927	2.0504082	down	hsa_circ_0003696	exonic	hsa-miR-93-3p	hsa-miR-329-5p	hsa-miR-501-5p	hsa-let-7i-5p	hsa-let-7b-5p
ASCRP3006441	0.009614777	2.0390738	down	hsa_circ_0089972	exonic	hsa-miR-129-5p	hsa-miR-3137	hsa-miR-1248	hsa-miR-4538	hsa-miR-6868-3p
ASCRP3003202	0.033416938	2.0352515	down	hsa_circ_0017675	exonic	hsa-miR-4799-3p	hsa-miR-153-5p	hsa-miR-223-3p	hsa-miR-4756-3p	hsa-miR-4453

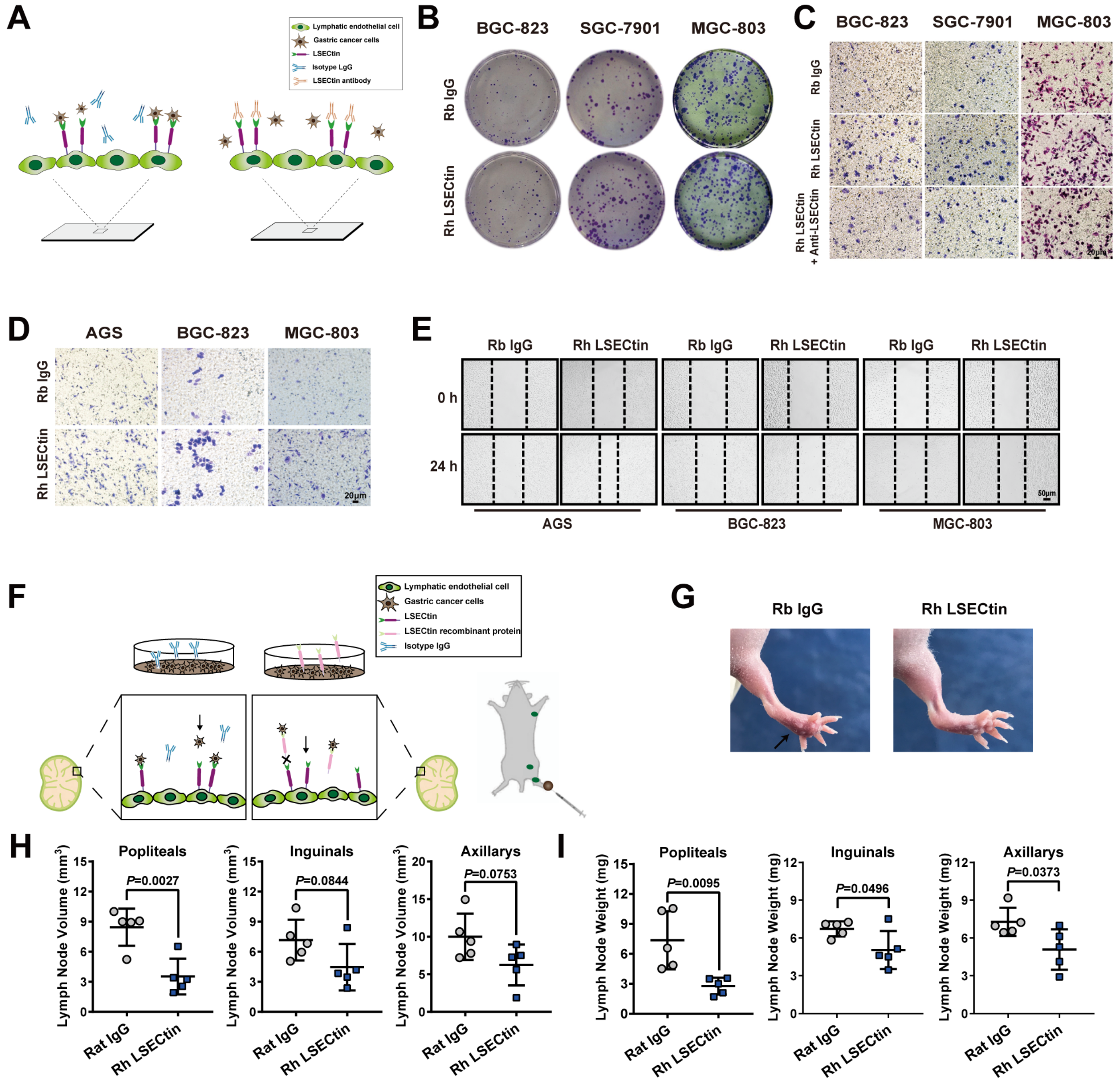

A
Lymphatic endothelial cell
Gastric cancer cells
LSECtin
Isotype LgG
LSECtin antibody
B
BGC-823
SGC-7901
MGC-803
Rb IgG
Rh LSECtin
C
BGC-823
SGC-7901
MGC-803
Rb IgG
Rh LSECtin
Rh LSECtin + Anti-LSECtin
20μm
D
AGS
BGC-823
MGC-803
Rb IgG
Rh LSECtin
20μm
E
Rb IgG
Rh LSECtin
0 h
24 h
50μm
AGS
BGC-823
MGC-803
F
Lymphatic endothelial cell
Gastric cancer cells
LSECtin
LSECtin recombinant protein
Isotype IgG
G
Rb IgG
Rh LSECtin
H
Popliteals
Inguinals
Axillarys
Lymph Node Volume (mm³)
P=0.0027
P=0.0844
P=0.0753
Rat IgG
Rh LSECtin
I
Popliteals
Inguinals
Axillarys
Lymph Node Weight (mg)
P=0.0095
P=0.0496
P=0.0373
Rat IgG
Rh LSECtin

Supplementary file 7: Figure S1. LSECtin positively regulates GC cell adhesion, proliferation, migration, and invasion in vitro and lymphatic metastasis in vivo. A The schematic overview of the adhesion experiment is shown**. B** Representative image of the clonogenic assay after cells were treated with the LSECtin protein or control IgG. **C, D** The representative image of the Transwell assay after cells were treated with the LSECtin protein or control IgG. **E** The representative image of the wound healing assay after cells were treated with the LSECtin protein or control IgG. **F**. The schematic overview of the animal experiment is shown**. G** Representative images of the nude mouse model of lymph node metastasis. **H, I** The volume and weight of the lymph nodes were measured and assessed.

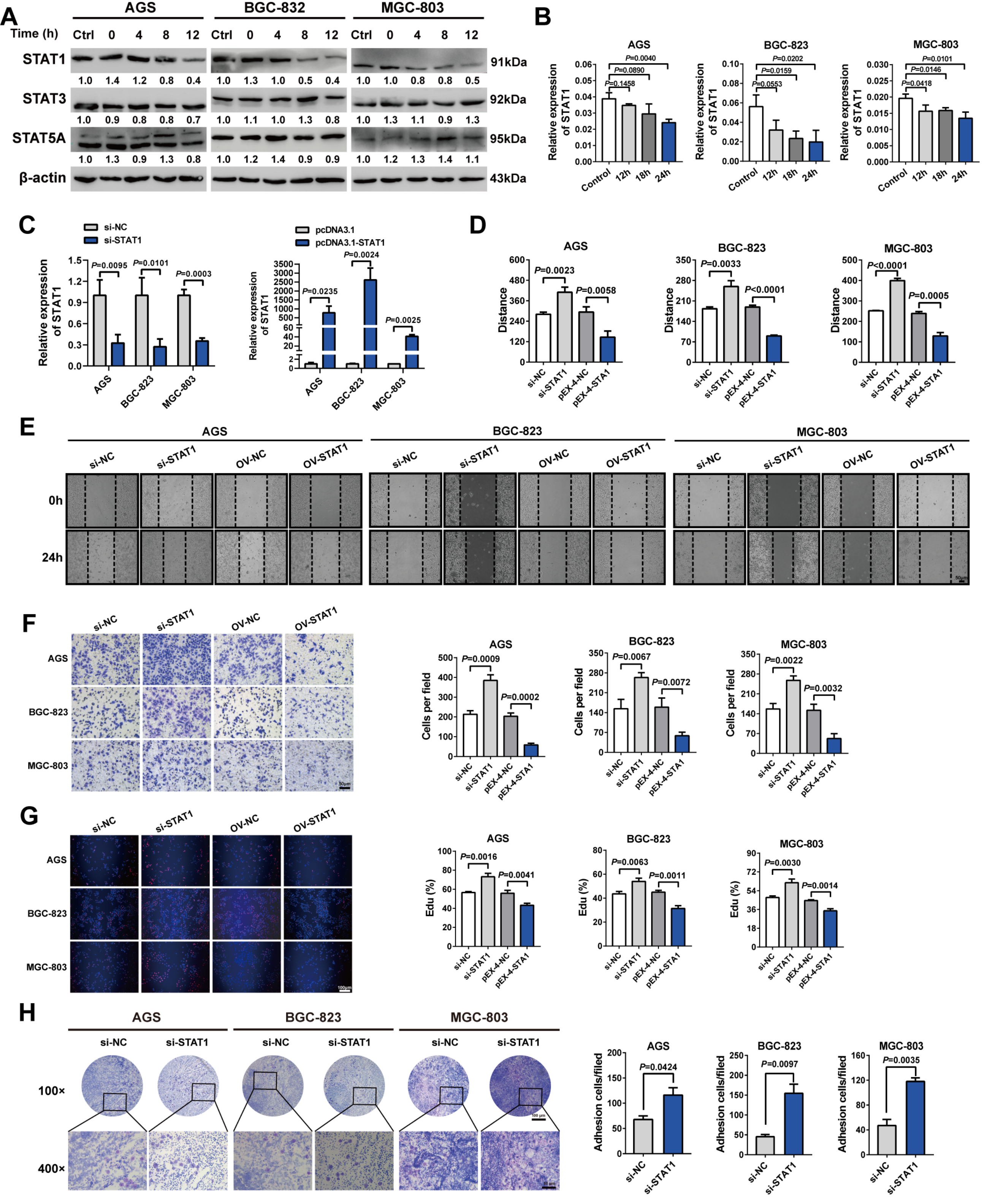

A
Time (h)
AGS
BGC-832
MGC-803
Ctrl 0 4 8 12
STAT1
1.0 1.4 1.2 0.8 0.4
1.0 1.3 1.0 0.5 0.4
1.0 1.0 0.8 0.8 0.5
91kDa
STAT3
1.0 0.9 0.8 0.8 0.7
1.0 1.1 1.0 1.3 0.8
1.0 1.3 1.1 0.9 1.3
92kDa
STAT5A
1.0 1.3 0.9 1.3 0.8
1.0 1.2 1.4 0.9 0.9
1.0 1.2 1.3 1.4 1.1
95kDa
β-actin
43kDa
B
AGS
BGC-823
MGC-803
Relative expression of STAT1
P=0.0040
P=0.0890
P=0.1458
P=0.0202
P=0.0159
P=0.0553
P=0.0101
P=0.0146
P=0.0418
Control 12h 18h 24h
C
si-NC
si-STAT1
Relative expression of STAT1
P=0.0095
P=0.0101
P=0.0003
pcDNA3.1
pcDNA3.1-STAT1
P=0.0235
P=0.0024
P=0.0025
AGS BGC-823 MGC-803
D
AGS
BGC-823
MGC-803
Distance
P=0.0023
P=0.0058
P=0.0033
P<0.0001
P<0.0001
P=0.0005
si-NC si-STAT1 pEX-4-NC pEX-4-STA1
E
AGS
BGC-823
MGC-803
si-NC si-STAT1 OV-NC OV-STAT1
0h
24h
F
si-NC si-STAT1 OV-NC OV-STAT1
AGS
BGC-823
MGC-803
Cells per field
P=0.0009
P=0.0002
P=0.0067
P=0.0072
P=0.0022
P=0.0032
si-NC si-STAT1 pEX-4-NC pEX-4-STA1
G
si-NC si-STAT1 OV-NC OV-STAT1
AGS
BGC-823
MGC-803
Edu (%)
P=0.0016
P=0.0041
P=0.0063
P=0.0011
P=0.0030
P=0.0014
si-NC si-STAT1 pEX-4-NC pEX-4-STA1
H
AGS
BGC-823
MGC-803
si-NC si-STAT1
100×
400×
Adhesion cells/filed
P=0.0424
P=0.0097
P=0.0035
si-NC si-STAT1

Supplementary file 8: Figure S2. STAT1 down-regulated by LSECtin in GC cells. A The expression of tumor-related STATs in gastric cancer cells treated with 5μg/ml LSECtin protein (0 h, 4 h, 8 h and 12 h) were detected by western blot, controls treated with isotype IgG for 12 h. **B** The downregulation of STAT1 mRNA levels induced by 1 μg/ml LSECtin protein in GC cell lines was detected by qRT-PCR. **C** QPCR confirmed STAT1 expression level upon knockdown or overexpression in GC cells. **D, E** The migration ability of the STAT1 knockdown and overexpression cells was measured via Wound healing assays. **F** The migration ability of the STAT1 knockdown and overexpression cells was measured by the Transwell migration assays. **G** The proliferation ability of the STAT1 knockdown and overexpression cells was measured by the EdU experiments. **H** Adhesion between GC cells and frozen mouse lymph node sections was detected in vitro by hemoxylin and eosin (H&E) staining Error bars indicate standard deviation (n = 3). Data, the means ± SD.

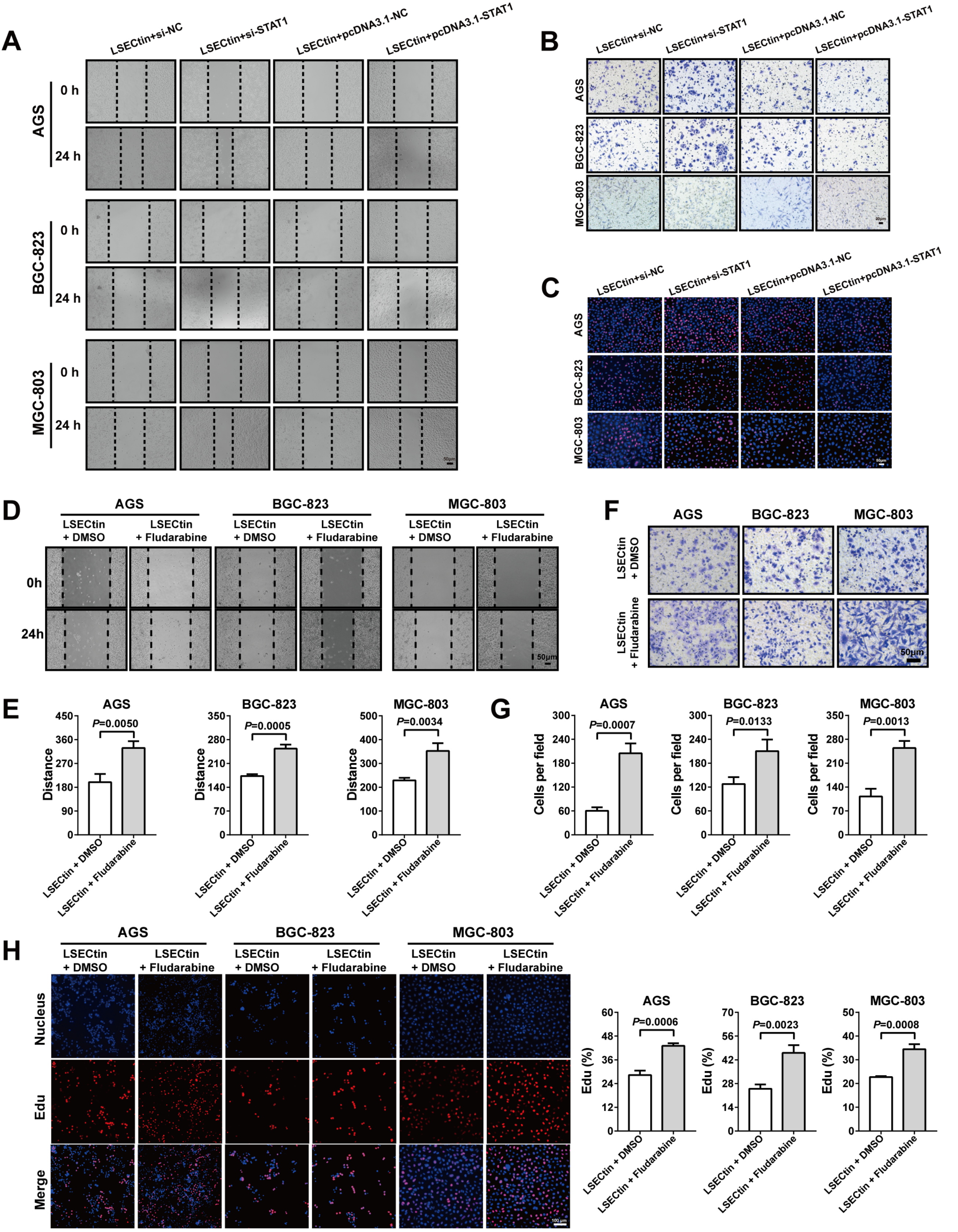
A
LSECtin+si-NC
LSECtin+si-STAT1
LSECtin+pcDNA3.1-NC
LSECtin+pcDNA3.1-STAT1
AGS
BGC-823
MGC-803
0 h
24 h
B
C
D
LSECtin + DMSO
LSECtin + Fludarabine
0h
24h
50μm
E
Distance
P=0.0050
P=0.0005
P=0.0034
F
G
Cells per field
P=0.0007
P=0.0133
P=0.0013
H
Nucleus
Edu
Merge
Edu (%)
P=0.0006
P=0.0023
P=0.0008

Supplementary file 9: Figure S3. STAT1 down-regulated by LSECtin is involved in GC adhension and migration.

A, B Representative images showing effect of different combination treatments on GC cells migration using Wound healing assays (A) and Transwell migration (B) assay. **C** Representative images showing effect of different combination treatments on GC cells proliferation using EdU experiments. **D, E** Wound healing assays showed that LSECtin enhanced GC cells migration treated with fludarabine. **F, G** Transwell migration assays showed that LSECtin enhanced GC cells migration treated with fludarabine. **H** EdU experiments showed that LSECtin enhanced GC cells proliferation treated with fludarabine. Error bars indicate standard deviation (n=3). The representative image of the Transwell assay Data, the means ± SD.

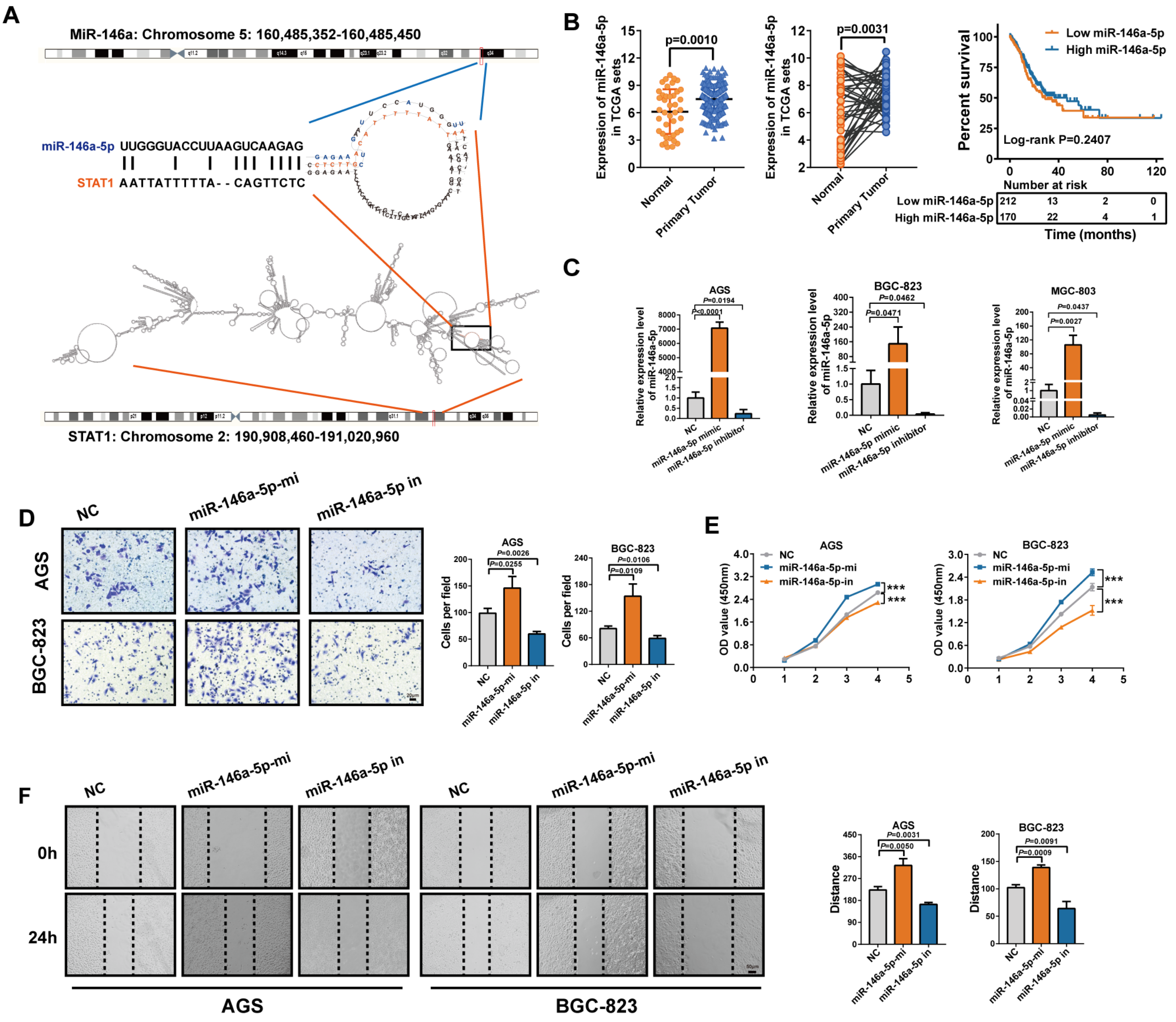
A
MiR-146a: Chromosome 5: 160,485,352-160,485,450
miR-146a-5p UUGGGUACCUUAAGUCAAGAG
STAT1 AATTATTTTTA- - CAGTTCTC
STAT1: Chromosome 2: 190,908,460-191,020,960
B
p=0.0010
Expression of miR-146a-5p in TCGA sets
Normal
Primary Tumor
p=0.0031
Low miR-146a-5p
High miR-146a-5p
Percent survival
Log-rank P=0.2407
Number at risk
Low miR-146a-5p 212 13 2 0
High miR-146a-5p 170 22 4 1
Time (months)
C
AGS
BGC-823
MGC-803
Relative expression level of miR-146a-5p
P=0.0194
P<0.0001
P=0.0462
P=0.0471
P=0.0437
P=0.0027
NC
miR-146a-5p mimic
miR-146a-5p inhibitor
D
NC
miR-146a-5p-mi
miR-146a-5p in
AGS
BGC-823
Cells per field
P=0.0026
P=0.0255
P=0.0106
P=0.0109
E
AGS
BGC-823
NC
miR-146a-5p-mi
miR-146a-5p-in
OD value (450nm)
***
F
0h
24h
Distance
P=0.0031
P=0.0050
P=0.0091
P=0.0009

Supplementary file 10: Figure S4. Examination of miR-146a-5p/STAT1 axis in gastric cancer. **A** Secondary structure of the specific miR-146a-5p-binding site and its flank regions in the STAT1 3'-UTR. **B** MiR-146a-5p expression levels in 415 cases of gasric cancer and 35 cases of normal tissue (left), miR-146a-5p expression levels in 32 tumor and matched normal samples of gastric cancer patients (right). **C** Gastric cancer cells were transfected with miR-146a-5p mimics, miR-146a-5p inhibitor or miR-NC, and the overexpression or knockdown of miR-146a-5p was investigated by qPCR. **D** The effect of miR-146a-5p on the migration of human gasric cancer cells assessed by transwell assays, the statistical views of transwell migration assays. **E** The cell viability was detected with miR-146a-5p mimics, miR-146a-5p inhibitor or miR-NC in gastric cancer cell lines by CCK8 assay. **F** The effect of miR-146a-5p on the migration of human gasric cancer cells assessed by wound healing assays. Error bars indicate standard deviation (n=3). Data, the means ± SD.

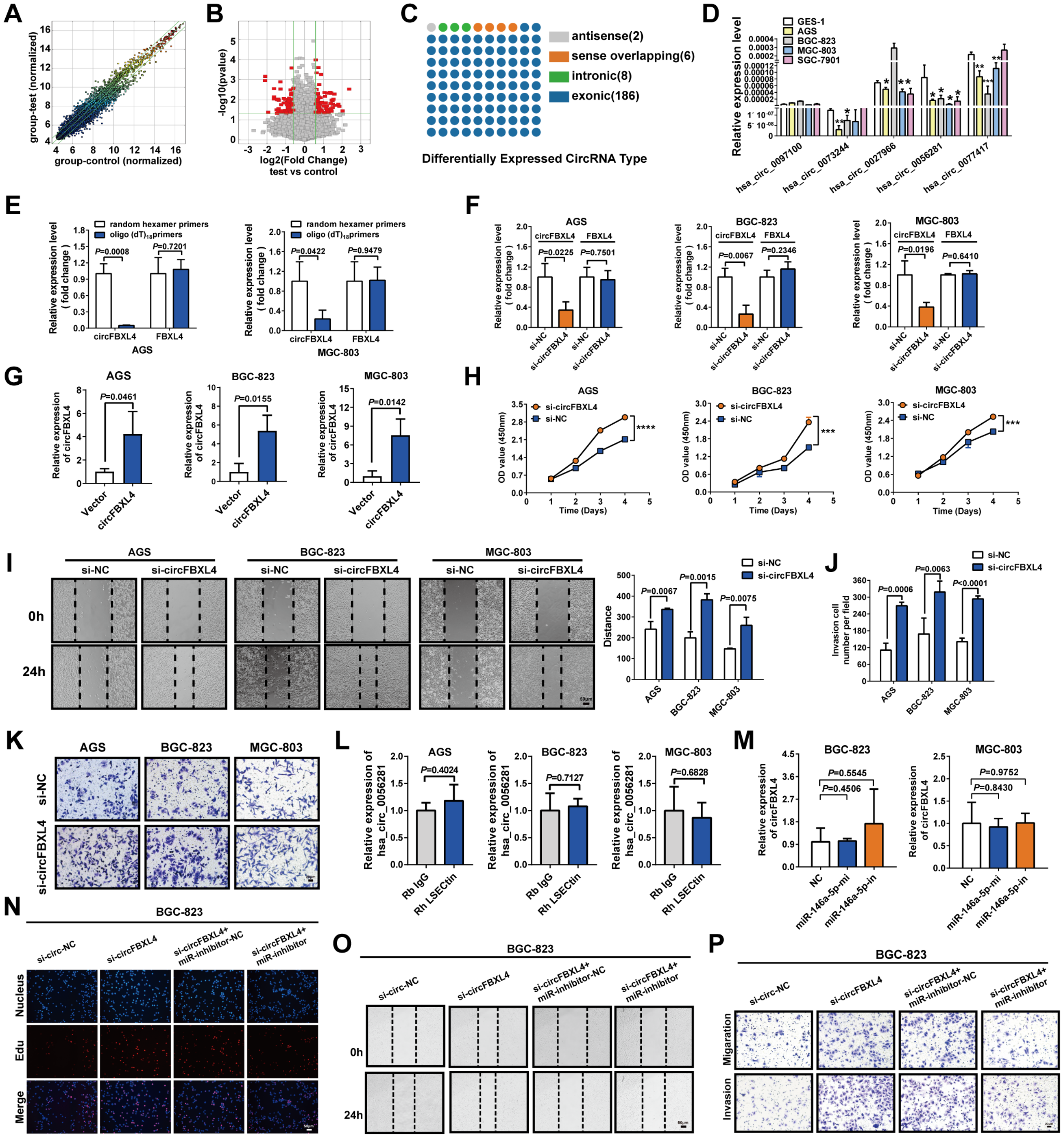
A
group-test (normalized)
group-control (normalized)
B
-log10(pvalue)
log2(Fold Change)
test vs control
C
antisense(2)
sense overlapping(6)
intronic(8)
exonic(186)
Differentially Expressed CircRNA Type
D
Relative expression level
GES-1
AGS
BGC-823
MGC-803
SGC-7901
hsa_circ_0097100
hsa_circ_0073244
hsa_circ_0027966
hsa_circ_0056281
hsa_circ_0077417
E
random hexamer primers
oligo (dT)18primers
Relative expression level (fold change)
P=0.0008
P=0.7201
circFBXL4
FBXL4
AGS
P=0.0422
P=0.9479
MGC-803
F
AGS
BGC-823
MGC-803
P=0.0225
P=0.7501
P=0.0067
P=0.2346
P=0.0196
P=0.6410
si-NC
si-circFBXL4
G
Relative expression of circFBXL4
P=0.0461
P=0.0155
P=0.0142
Vector
H
OD value (450nm)
Time (Days)
I
0h
24h
Distance
P=0.0067
P=0.0015
P=0.0075
J
Invasion cell number per field
P=0.0006
P=0.0063
P<0.0001
K
L
Relative expression of hsa_circ_0056281
P=0.4024
P=0.7127
P=0.6828
Rb IgG
Rh LSECtin
M
P=0.5545
P=0.4506
P=0.9752
P=0.8430
NC
miR-146a-5p-mi
miR-146a-5p-in
N
si-circ-NC
si-circFBXL4+ miR-inhibitor-NC
si-circFBXL4+ miR-inhibitor
Nucleus
Edu
Merge
O
P
Migaration
Invasion

Supplementary file 11: Figure S5. CircFBXL4/miR-146a-5p/STAT1 axis constructed in GC. **A** The scatter plot indicates the variation in circRNA expression between GC and normal stomach samples (log2 scaled). **B** Volcano plots showing the fold-change values and P-values of the array data. The red point represents significantly upregulated or downregulated circRNAs. **C** CircRNAs were classified in four types. **D** CircRNA expression in GC cells and normal gastric epithelial cells. **E** Oligo (dT) $_{18}$ primers and random primers were used for reverse transcription experiments. The relative RNA levels of circFBXL4 were analyzed by qRT-PCR. **F, G** qPCR experiments show overexpression or knockdown efficiency of circFBXL4. **H** Assessment of the proliferation of GC cells transfected with control or circFBXL4 siRNA by CCK-8 assay. **I-K** Wound healing assay and Transwell migration experiments showed that knockdown of circFBXL4 promoted the migration of gastric cancer cell lines AGS, BGC-823 and MGC-803. **L** LSECtin did not affect hsa_circ_0056281 expression. **M** The circFBXL4 expression after miR-146a-5p overexpression or silencing in GC cells was tested by qRT-PCR. **N** Representative images of EdU assays indicated that miR-146a-5p inhibitors abolished the promotion of proliferation induced by circFBXL4 knockdown. **N** Representative images of Wound healing assays showed that miR-146a-5p inhibitors suppressed the migration induced by circFBXL4 knockdown. **O**, **P** Representative images of Transwell assays showed that miR-146a-5p inhibitors suppressed the migration and invasion induced by circFBXL4 knockdown. Error bars indicate standard deviation (n = 3). Data, the means ± SD.

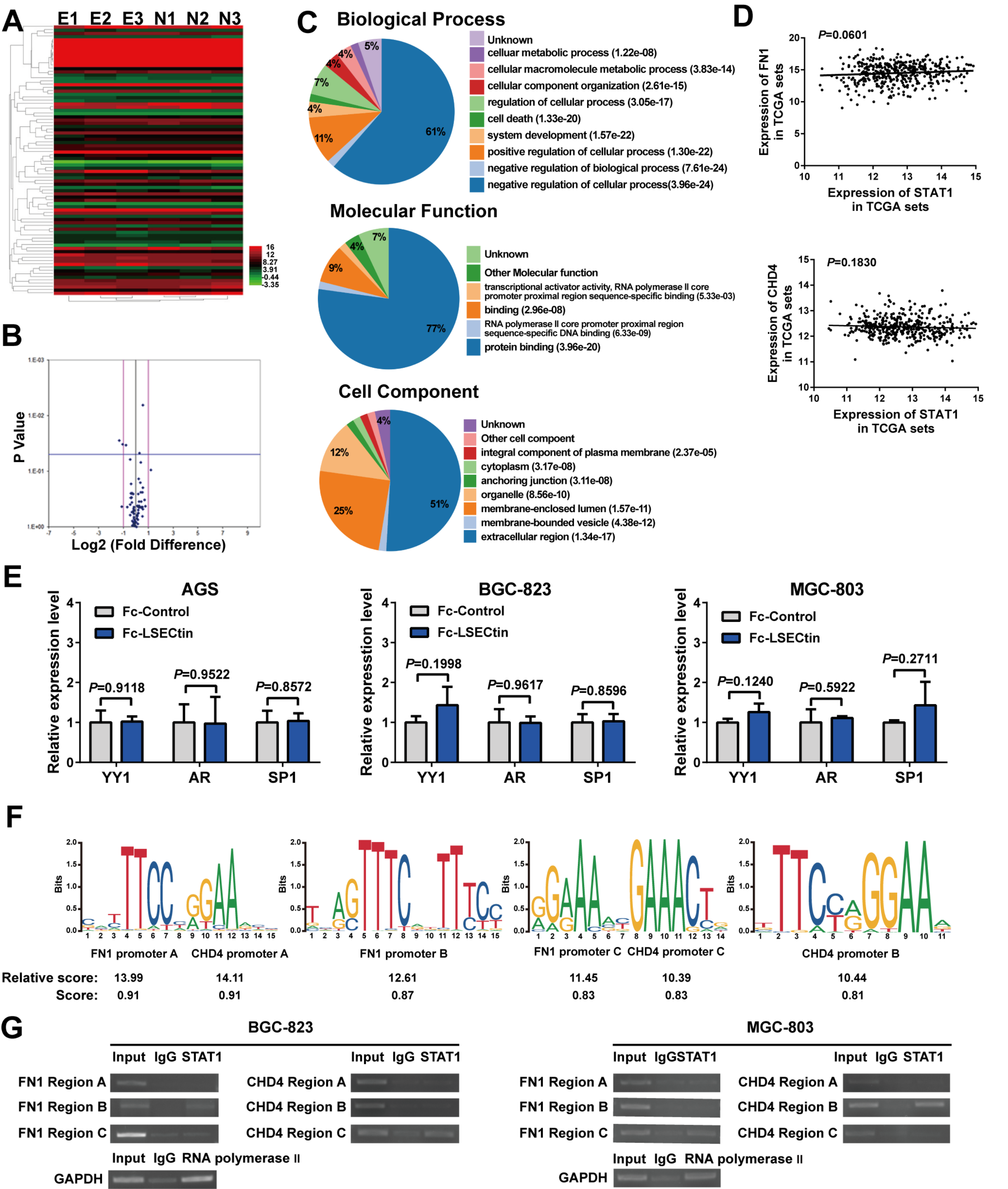

A
E1 E2 E3 N1 N2 N3
16
12
8.27
3.91
-0.44
-3.35
B
P Value
Log2 (Fold Difference)
C
Biological Process
Unknown
celluar metabolic process (1.22e-08)
cellular macromolecule metabolic process (3.83e-14)
cellular component organization (2.61e-15)
regulation of cellular process (3.05e-17)
cell death (1.33e-20)
system development (1.57e-22)
positive regulation of cellular process (1.30e-22)
negative regulation of biological process (7.61e-24)
negative regulation of cellular process(3.96e-24)
61%
11%
7%
5%
4%
4%
4%
Molecular Function
Unknown
Other Molecular function
transcriptional activator activity, RNA polymerase II core promoter proximal region sequence-specific binding (5.33e-03)
binding (2.96e-08)
RNA polymerase II core promoter proximal region sequence-specific DNA binding (6.33e-09)
protein binding (3.96e-20)
77%
9%
7%
4%
Cell Component
Unknown
Other cell compoent
integral component of plasma membrane (2.37e-05)
cytoplasm (3.17e-08)
anchoring junction (3.11e-08)
organelle (8.56e-10)
membrane-enclosed lumen (1.57e-11)
membrane-bounded vesicle (4.38e-12)
extracellular region (1.34e-17)
51%
25%
12%
4%
D
P=0.0601
Expression of FN1 in TCGA sets
Expression of STAT1 in TCGA sets
P=0.1830
Expression of CHD4 in TCGA sets
Expression of STAT1 in TCGA sets
E
AGS
Fc-Control
Fc-LSECtin
P=0.9118
P=0.9522
P=0.8572
Relative expression level
YY1 AR SP1
BGC-823
P=0.1998
P=0.9617
P=0.8596
Relative expresstion level
MGC-803
P=0.1240
P=0.5922
P=0.2711
F
Bits
FN1 promoter A CHD4 promoter A
FN1 promoter B
FN1 promoter C CHD4 promoter C
CHD4 promoter B
Relative score: 13.99 14.11 12.61 11.45 10.39 10.44
Score: 0.91 0.91 0.87 0.83 0.83 0.81
G
BGC-823
MGC-803
Input IgG STAT1
FN1 Region A
FN1 Region B
FN1 Region C
CHD4 Region A
CHD4 Region B
CHD4 Region C
Input IgG RNA polymerase II
GAPDH

Supplementary file 12: Figure S6. LSECtin induced FN1 and CHD4 expression through STAT1 transcriptional regulation. **A** Hierarchical clustering revealed differentially expressed genes stimulated with 1 μg/ml LSECtin protein in MGC-803 cells. **B** Volcano plots suggesting that the fold-change values (log2 scaled) and P-values of the array data. **C** The pie chart shows the biological process, cell component and molecular function categories of differentially expressed genes. **D** The association of FN1 and/or CHD4 expression and STAT1 from the publicly available TCGA. **E** Differential expression of YY1, AR and SP1 in gastric cancer cells treated with 1μg/ml LSECtin protein (24 h) were detected by Qpcr. **F** The schematic overview of the STAT1 motifs was provided. **G** chromatin immunoprecipitation (ChIP) assay confirmed FN1 and CHD4 promoter regions relative enrichment by the STAT1 antibody. Error bars indicate standard deviation (n = 3). Data, mean ± SD.
