# Supplementary material for: Novel roles of LSECtin in gastric cancer cell adhesion, migration, invasion, and lymphatic metastasis
Source: Cell Death Dis. 2022 Jul 11;13(7):593. doi: 10.1038/s41419-022-05026-x (PMC9276708; doi:10.1038/s41419-022-05026-x)
Supplement: Supplementary file 4 — cddis-author-contribution-form [file 41419_2022_5026_MOESM4_ESM.pdf]

DECLARATION OF CONTRIBUTIONS TO ARTICLE

ADMC

Manuscript Number: CDDIS-21-1634

Journal Name: *Cell Death & Disease* (the 'Journal')

Proposed Title of the Contribution:

Novel roles of LSECtin in gastric cancer cell adhesion, migration, invasion, and lymphat ic metastasis (the 'Contribution)

Author(s):

Yinan Zhang, Zhen Feng, Yue Xu, Sufen Jiang, Qianshi Zhang, Zhenyu Zhang, Keyong Wang, Xiaomeng Li, Lijie Xu, Menglang Yuan, Zihao Chen, Jingyi Cui, Han Wu, Yina Gao, Wei Wei, Bo Wang, Yunfei Zuo and Shuangyi Ren (the 'Authors')

For all *CDDis* articles, each person named as an author in the published version must be able to show he or she has contributed substantially to the article.

Authorship credit should be based on 1) substantial contributions to conception and design, acquisition of data, or analysis and interpretation of data; 2) drafting the article or revising it critically for important intellectual content; and 3) final approval of the version to be published. Authors should meet conditions 1, 2 and 3.

Any person who cannot be shown to have made a substantial contribution to the article cannot be listed as an author in the final version. The name of any person who is deemed to have made a minor contribution can, however, appear in the Acknowledgments section of the article.

Please complete the table below to indicate the contributions of all named authors to the manuscript.

Author Full Name:	Specification of Contribution to the Manuscript:
Yinan Zhang	designed, performed the experiment and wrote manuscript.
Zhen Feng	collected tissue samples and clinical data
YueXu	helped write manuscript and performed the experiment
Sufen Jiang	provided material support and data collection
Qianshi Zhang	collected tissue samples and gave help in animal experiments
Zhenyu Zhang	gave help in animal experiments
Keyong Wang	experiments and data collection
Xiaomeng Li	cell culture and treatment, collected tissue samples for Chip
Lijie Xu	experiments and data collection
Menglang Yuan	provided help in design, gave help in cell culture and treatment
Zihao Chen	provided technical and material support
Jingyi Cui	provided technical support, gave help in cell culture and treatment
Han Wu	gave help in animal experiments and data collection

DECLARATION OF CONTRIBUTIONS TO ARTICLE

ADMC

Manuscript Number:	Journal Name:	
CDDIS-21-1634	*Cell Death & Disease*	(the 'Journal')

Proposed Title of the Contribution:

Novel roles of LSECtin in gastric cancer cell adhesion, migration, invasion, and lymphatic metastasis (the 'Contribution)

Author(s):

Yinan Zhang, Zhen Feng, Yue Xu, Sufen Jiang, Qianshi Zhang, Zhenyu Zhang, Keyong Wang, Xiaomeng Li, Lijie Xu, Menglang Yuan, Zihao Chen, Jingyi Cui, Han Wu, Yina Gao, Wei Wei, Bo Wang, Yunfei Zuo and Shuangyi Ren (the 'Authors')

For all *CDDis* articles, each person named as an author in the published version must be able to show he or she has contributed substantially to the article.

Authorship credit should be based on 1) substantial contributions to conception and design, acquisition of data, or analysis and interpretation of data; 2) drafting the article or revising it critically for important intellectual content; and 3) final approval of the version to be published. Authors should meet conditions 1, 2 and 3.

Any person who cannot be shown to have made a substantial contribution to the article cannot be listed as an author in the final version. The name of any person who is deemed to have made a minor contribution can, however, appear in the Acknowledgments section of the article.

Please complete the table below to indicate the contributions of all named authors to the manuscript.

Author Full Name:	Specification of Contribution to the Manuscript:
Yina Gao	gave help in animal experiments and data collection
Wei Wei	provided technical support, gave help in cell culture and treatment
Bo Wang	provided technical and material support
Yunfei Zuo	designed，revised the manuscript, approved the final version
Shuangyi Ren	designed，provided help in design and clinical support，revised the manuscript, approved the final version

Please complete the table below to indicate the contributions of all named authors to the figures.

Figure 1:

In Figure1, Keyong Wang generated the data and prepared panel A, B. Lijie Xu generated the data and prepared panel C. Yinan Zhang generated the data and prepared panel D,E. Zhen Feng collected clinical samples and clinical information,Yinan Zhang, Yina Gao and Sufen Jiang assembled the figure F. Zihao Chen helped generated information of panel F. Shuangyi Ren and Yunfei Zuo contributed to work supervision and design.

Figure 2:

In Figure2, Yinan Zhang generated the data and prepared panel A, B and G. Yina Gao and Sufen Jiang helped generated information,assembled the figure. Keyong Wang generated the data and prepared panel C-E. Lijie Xu and Yinan Zhang generated the data and prepared panel F. Yinan Zhang, Zhenyu Zhang, Han Wu and Yina Gao generated the data and prepared panel H-I, Qian Shi, Zihao Chen， Bo Wang and Sufen Jiang helped generated information of panel I. Sufen Jiang and Wei Wei gave help in cell culture and treatment. Shuangyi Ren and Yunfei Zuo contributed to work supervision, direction and design.

Figure 3:

In Figure3, Yinan Zhang and Yue Xu generated the data and prepared panel A, Yinan Zhang generated the data and prepared panel B. Yinan Zhang generated the data and prepared panel C-G, Sufen Jiang and Yina Gao helped generated information of panel E-G, Zhenyu Zhang, Han Wu and Bo Wang helped generated information,assembled the figure H. Yunfei Zuo contributed to work supervision, direction and design.

Figure 4:

In Figure4, Xiaomeng Li and Yinan Zhang collected clinical samples, Xiaomeng Li colleced to company for circRNA Chip. Yinan Zhang generated the data and prepared Figure4. Menglang Yuan helped generated information of panel B,C and K. Jingyi Cui helped generated information of panelG,J,K and provided technical support. Zihao Chen and Sufen Jiang helped generated information of panel J. Sufen Jiang and Yina Gao helped generated information of panel I, M-O. Shuangyi Ren and Yunfei Zuo contributed to work supervision, direction and design.

Figure 5:

In Figure5, Xiaomeng Li cell culture and treatment, collected samples to company for Chip. Yinan Zhang collected data for bioinformation and generated panel A, B. YueXu generated the data and prepared panel C, D,and G-J. Menglang Yuan helped generated information of panel J, provided technical support, Yinan Zhang involved in E,F,and K. assembled the figure with YueXu. Yunfei Zuo contributed to work supervision, direction and design.

Figure 6:

In Figure6, Yinan Zhang generated the data and prepared Figure6. Sufen Jiang helped generated information of Figure6. Han Wu helped generated information of panel F,H,and J-K. Zhenyu Zhang and Yina Gao helped generated information of panel F-I,J-K. Bo Wang provided technical support of panel I. Shuangyi Ren and Yunfei Zuo contributed to work supervision, direction and design.

Signed for and on behalf of the Author(s):	Print Name:	Date:
Shuangyi Ren	Shuangyi Ren	2022.1.4
