# Supplementary material for: Novel roles of LSECtin in gastric cancer cell adhesion, migration, invasion, and lymphatic metastasis
Source: Cell Death Dis. 2022 Jul 11;13(7):593. doi: 10.1038/s41419-022-05026-x (PMC9276708; doi:10.1038/s41419-022-05026-x)
Supplement: Supplementary file 5 — Original Data File [file 41419_2022_5026_MOESM5_ESM.pdf]

Fig 1

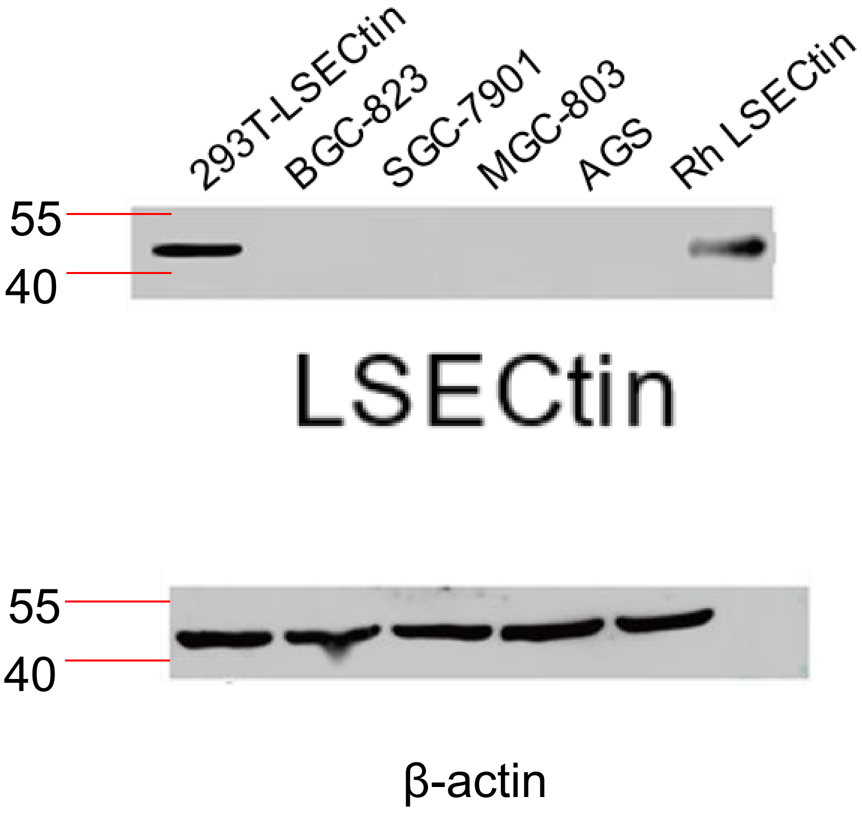
293T-LSECtin
BGC-823
SGC-7901
MGC-803
AGS
Rh LSECtin
55
40
LSECtin
55
40
β-actin

Fig 2

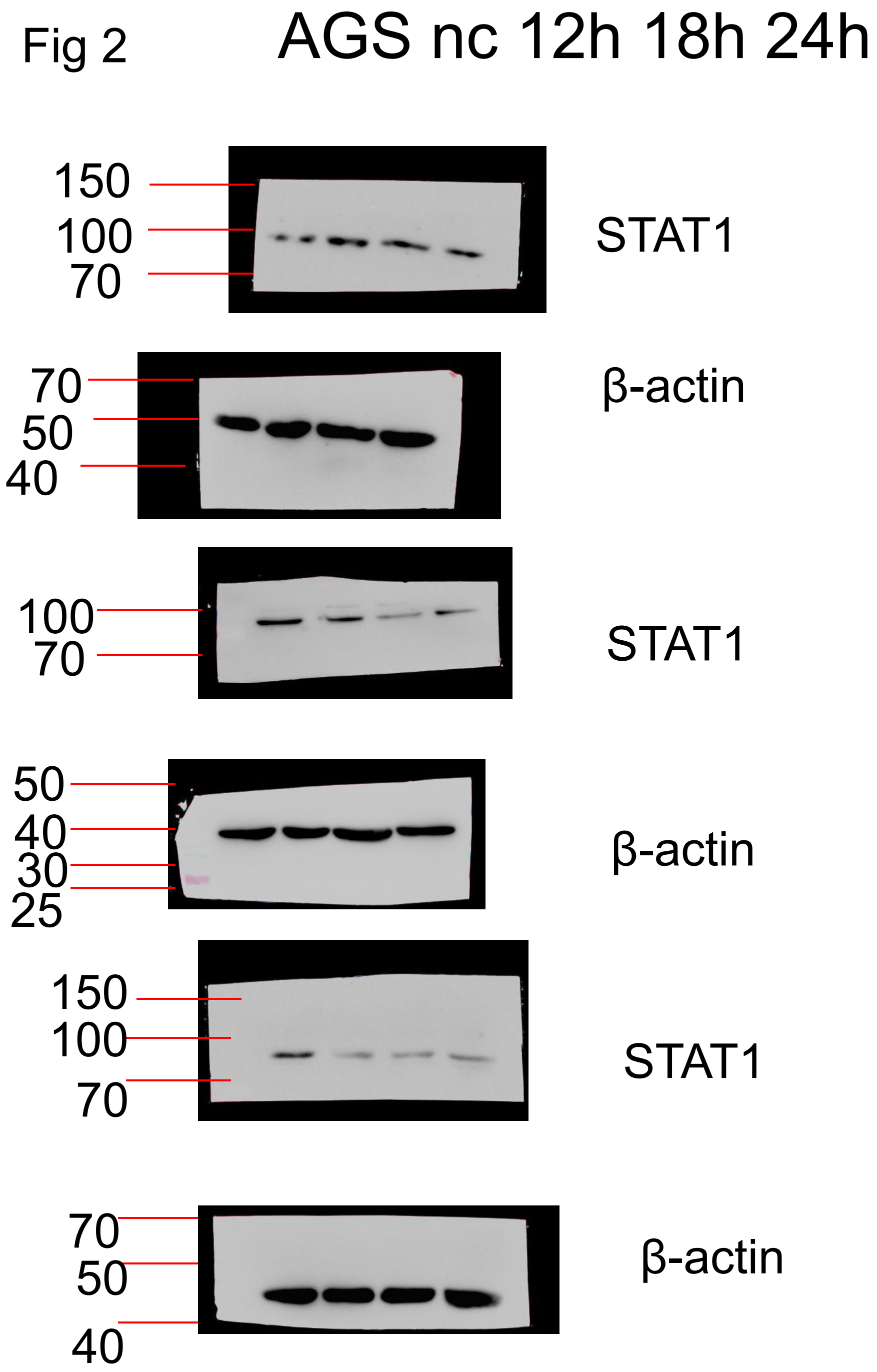
AGS nc 12h 18h 24h
150
100
70
STAT1
70
50
40
β-actin
100
70
STAT1
50
40
30
25
β-actin
150
100
70
STAT1
70
50
40
β-actin

Fig 2

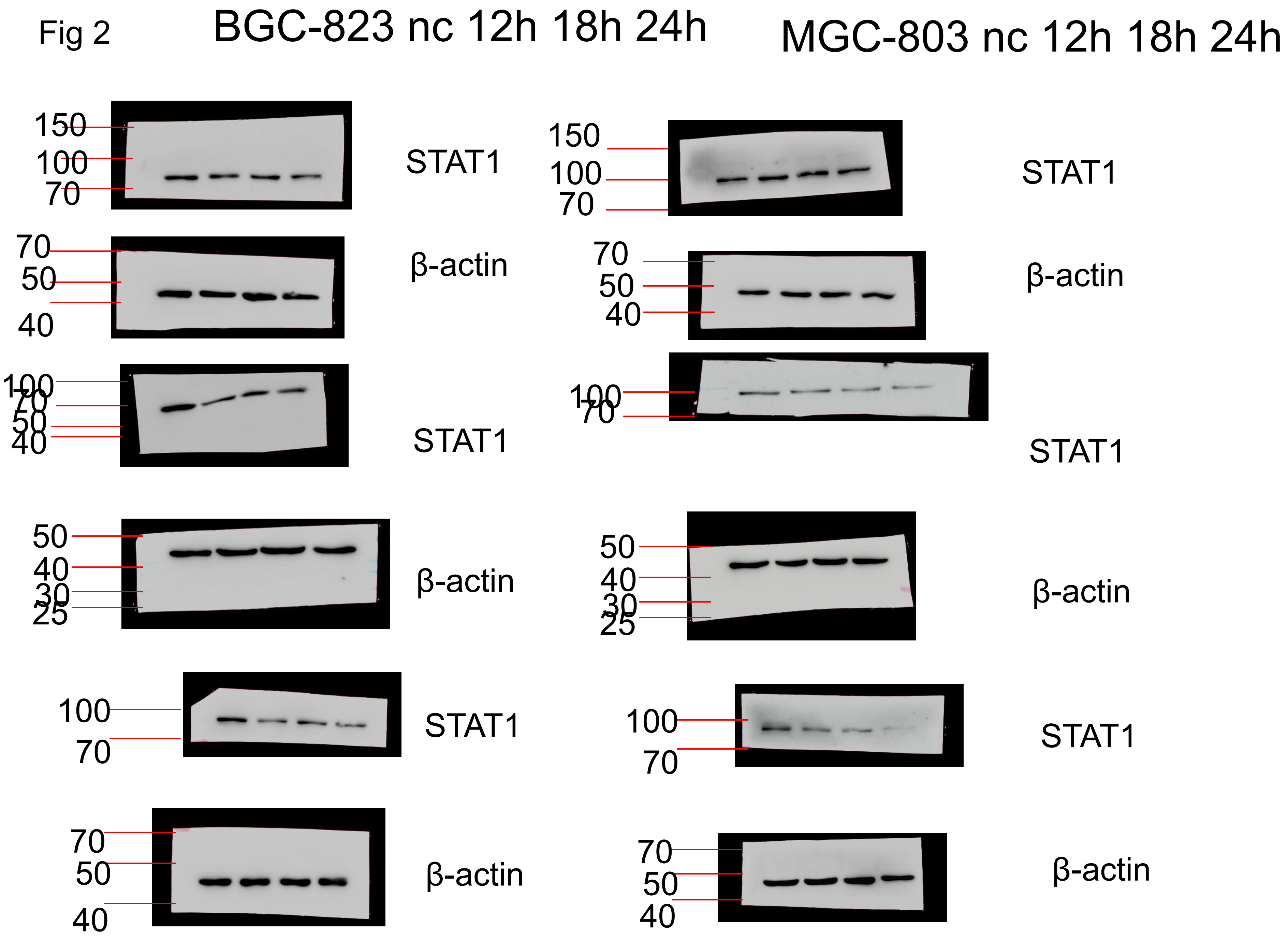
BGC-823 nc 12h 18h 24h
MGC-803 nc 12h 18h 24h
STAT1
β-actin
STAT1
β-actin
STAT1
β-actin
STAT1
β-actin
STAT1
β-actin
STAT1
β-actin

Fig 2

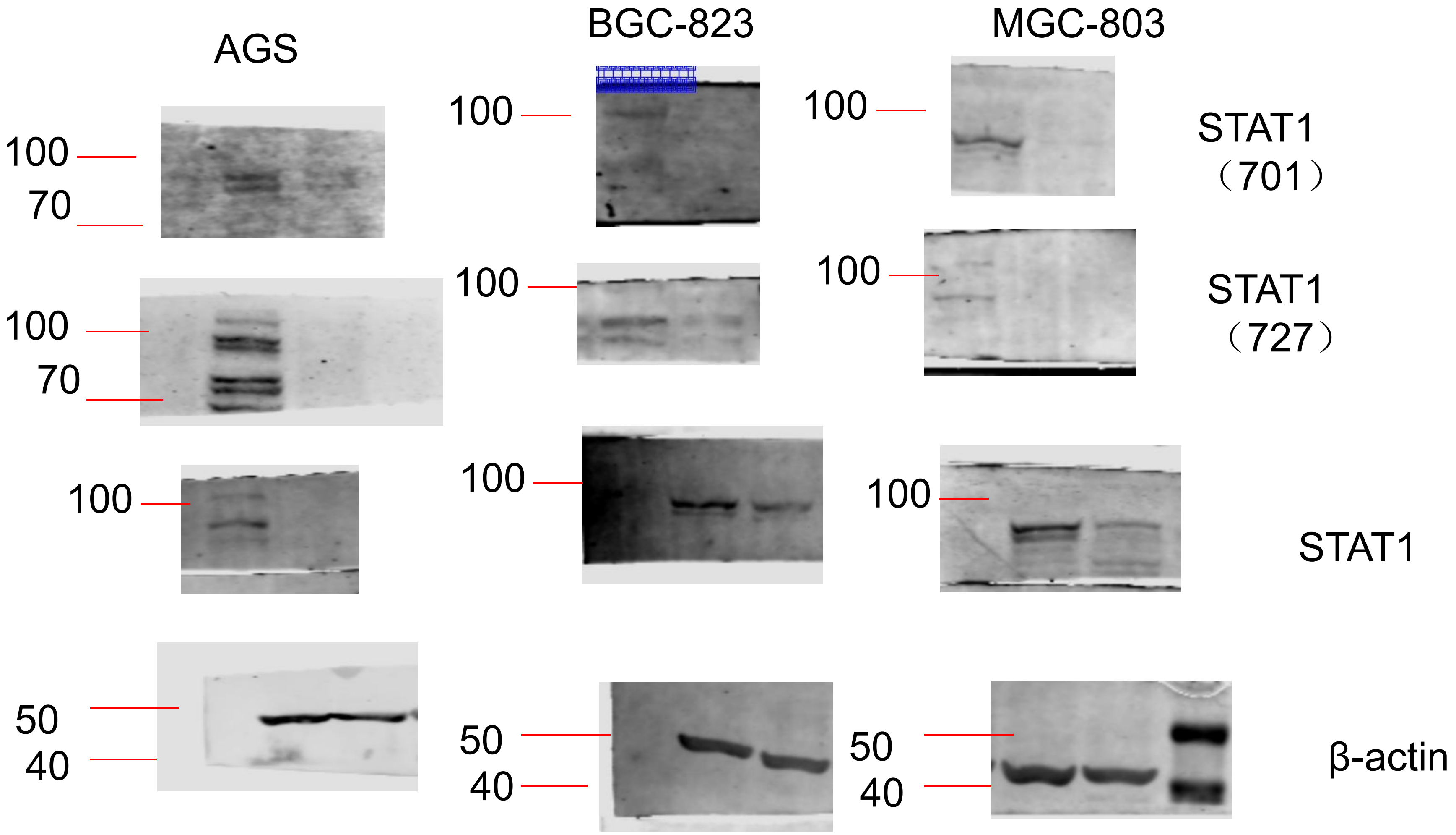
AGS
BGC-823
MGC-803
100
70
100
70
100
50
40
100
100
100
50
40
100
100
100
50
40
STAT1
（701）
STAT1
（727）
STAT1
β-actin

Fig4

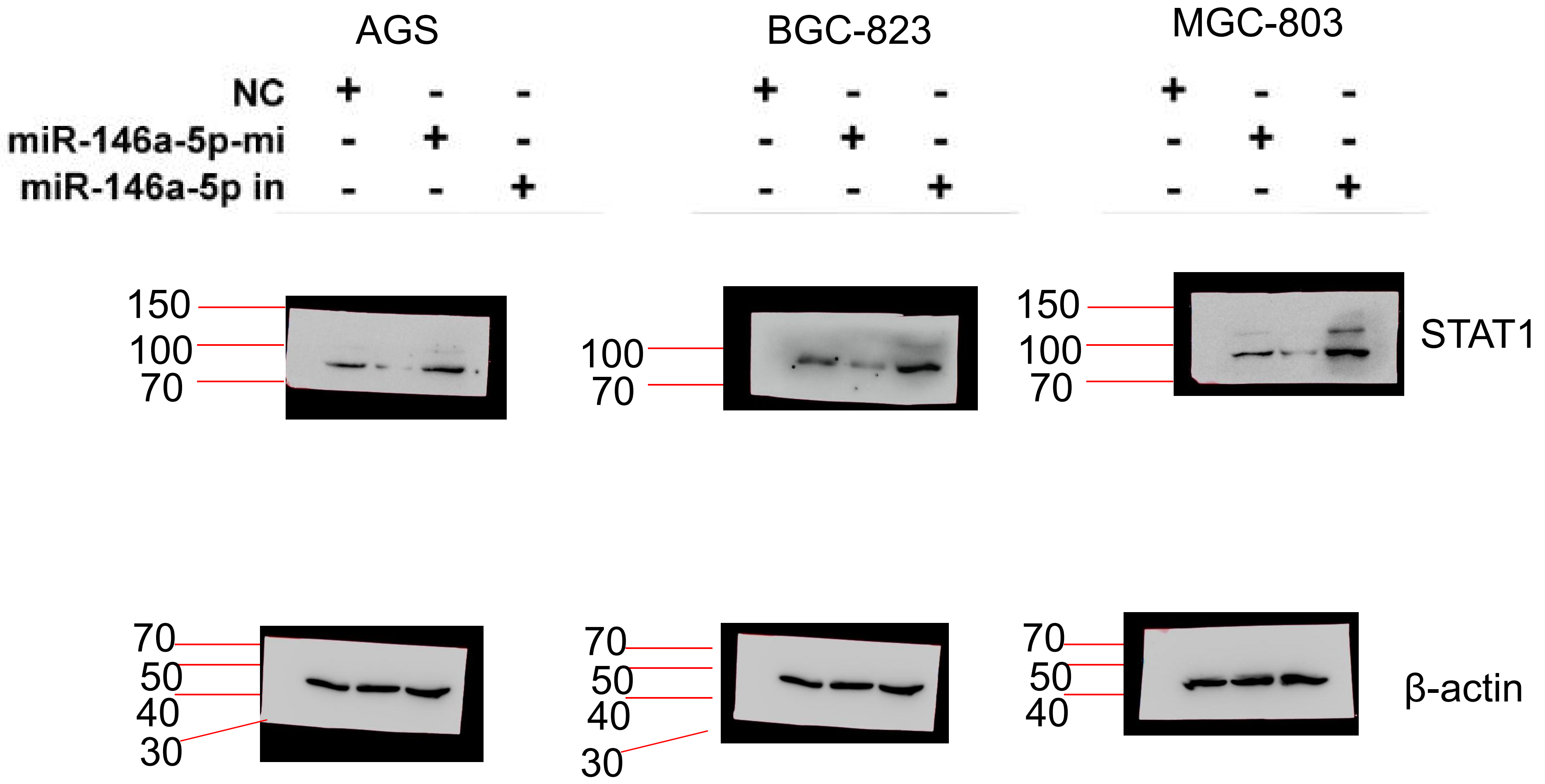
AGS
BGC-823
MGC-803
NC + - - + - - + - -
miR-146a-5p-mi - + - - + - - + -
miR-146a-5p in - - + - - + - - +
150
100
70
STAT1
70
50
40
30
β-actin

Fig 4

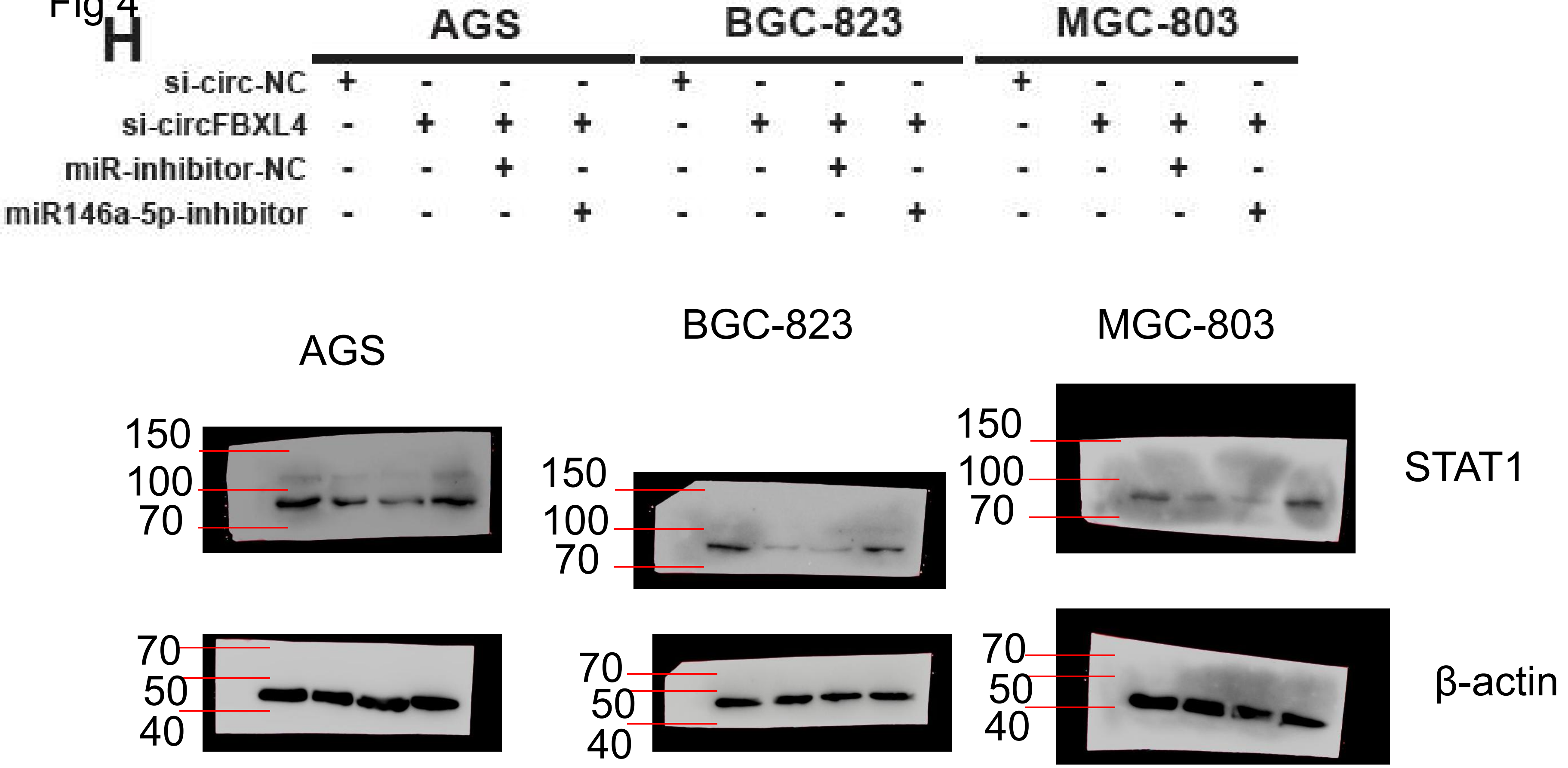
H
AGS
BGC-823
MGC-803
si-circ-NC + - - - + - - - + - - -
si-circFBXL4 - + + + - + + + - + + +
miR-inhibitor-NC - - + - - - + - - - + -
miR146a-5p-inhibitor - - - + - - - + - - - +
AGS
BGC-823
MGC-803
150
100
70
STAT1
70
50
40
β-actin

Fig 5

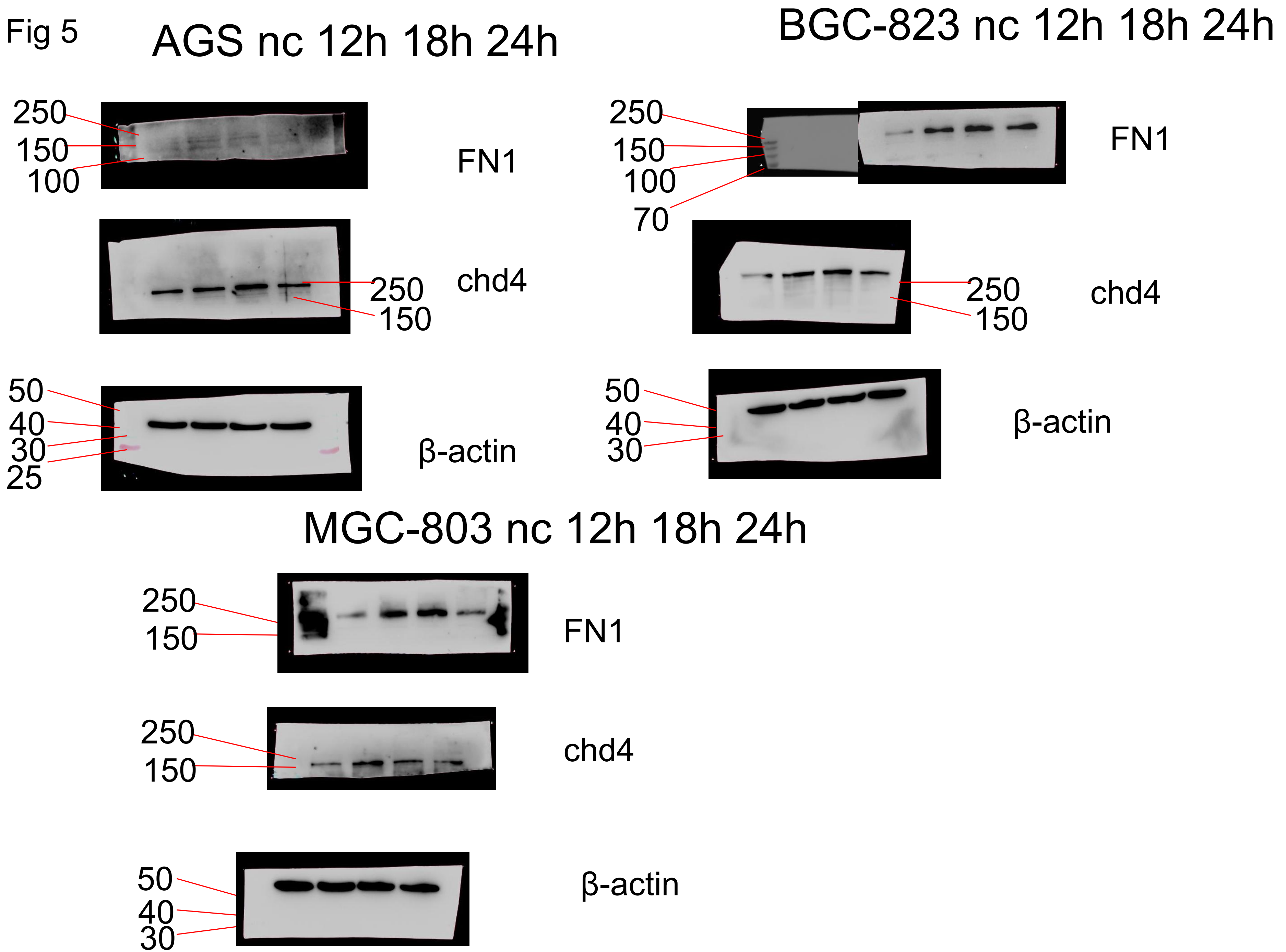
AGS nc 12h 18h 24h
250
150
100
FN1
chd4
250
150
50
40
30
25
β-actin
BGC-823 nc 12h 18h 24h
250
150
100
70
FN1
250
150
chd4
50
40
30
β-actin
MGC-803 nc 12h 18h 24h
250
150
FN1
250
150
chd4
50
40
30
β-actin

Fig 5

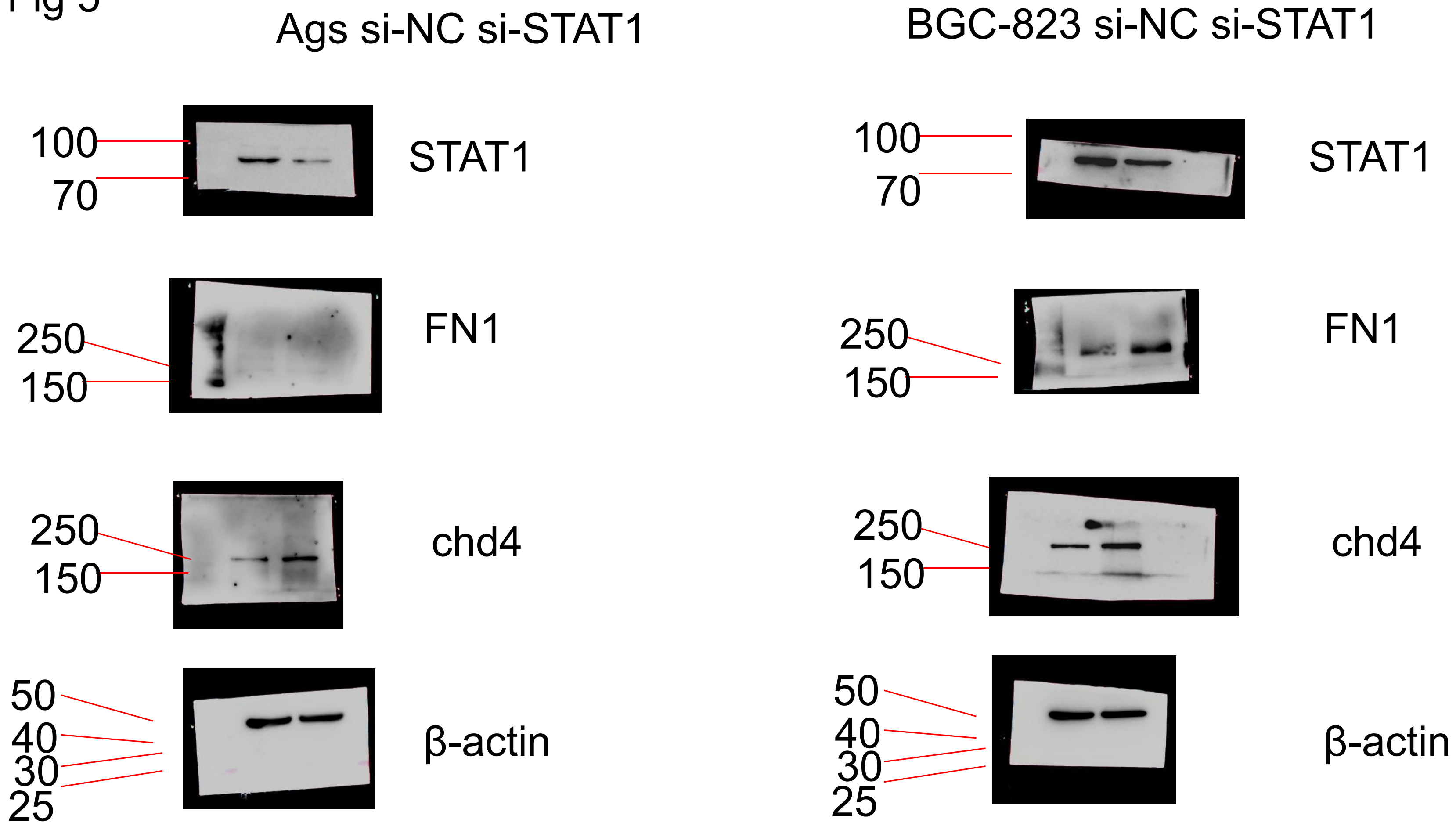

Ags si-NC si-STAT1
BGC-823 si-NC si-STAT1
100
70
STAT1
250
150
FN1
chd4
50
40
30
25
β-actin

Fig 5

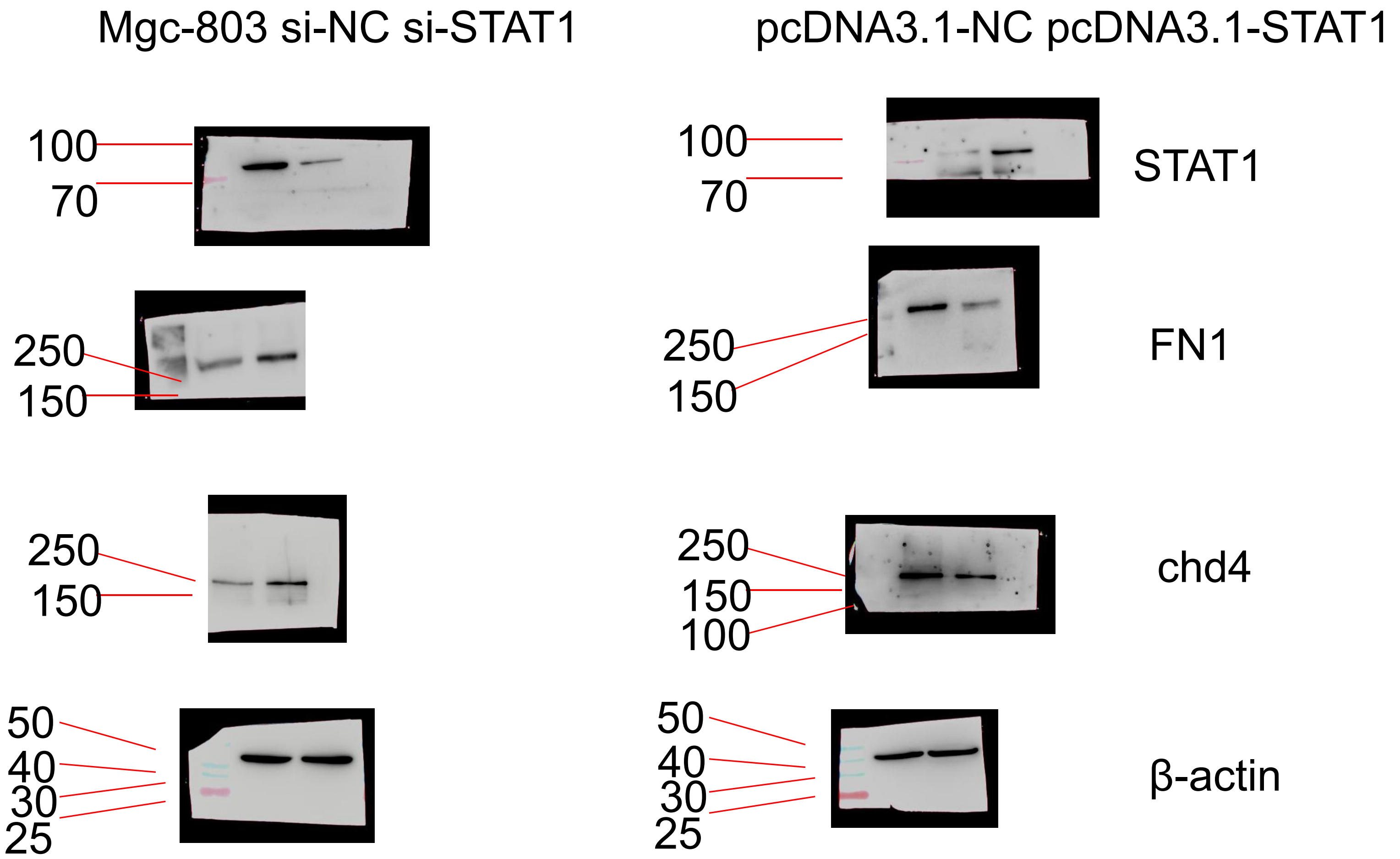
Mgc-803 si-NC si-STAT1
pcDNA3.1-NC pcDNA3.1-STAT1
100
70
STAT1
250
150
FN1
250
150
100
chd4
50
40
30
25
β-actin

Fig 5

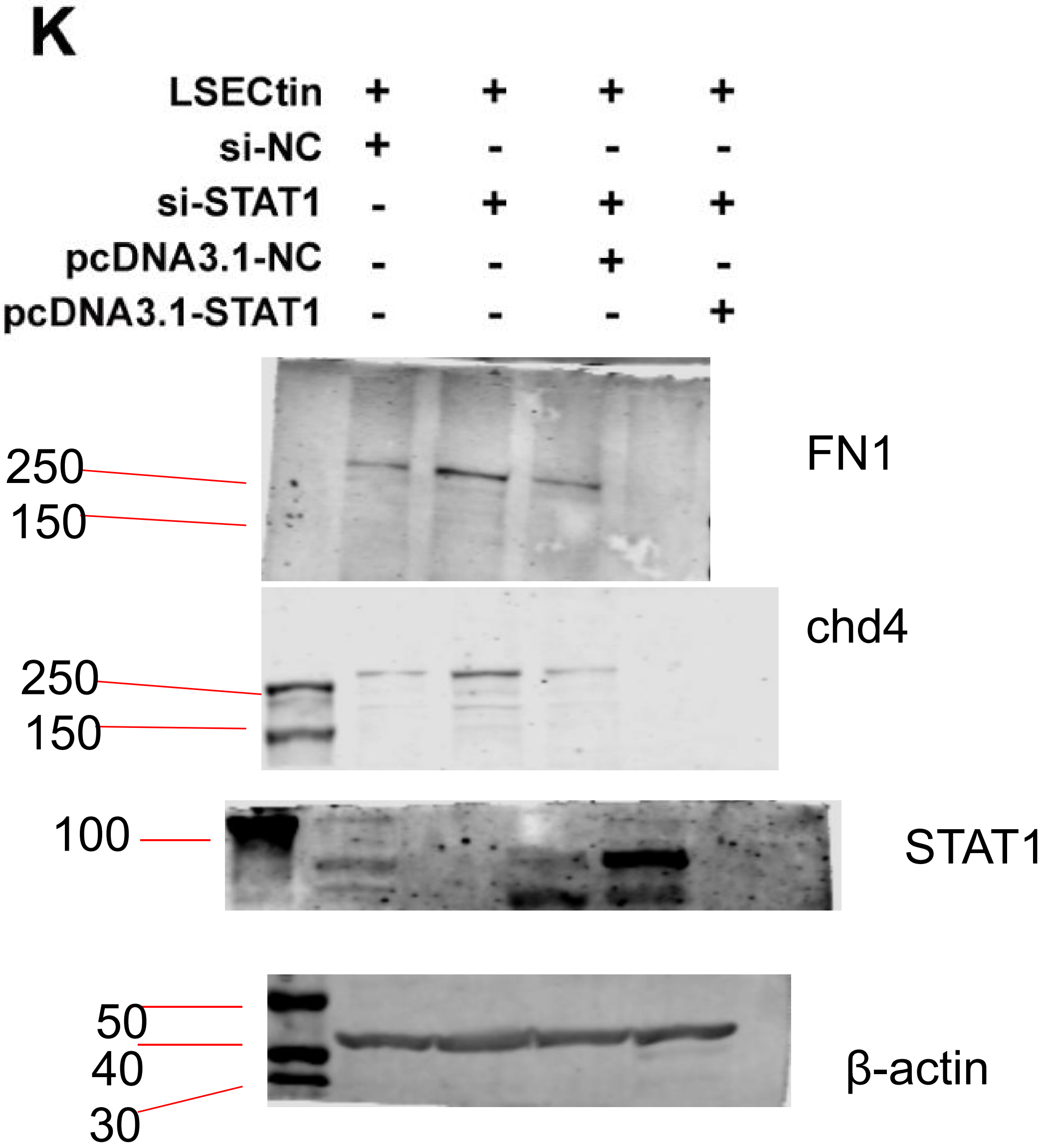
K
LSEctin + + + +
si-NC + - - -
si-STAT1 - + + +
pcDNA3.1-NC - - + -
pcDNA3.1-STAT1 - - - +
250
150
FN1
chd4
250
150
100
STAT1
50
40
30
β-actin

Fig 6

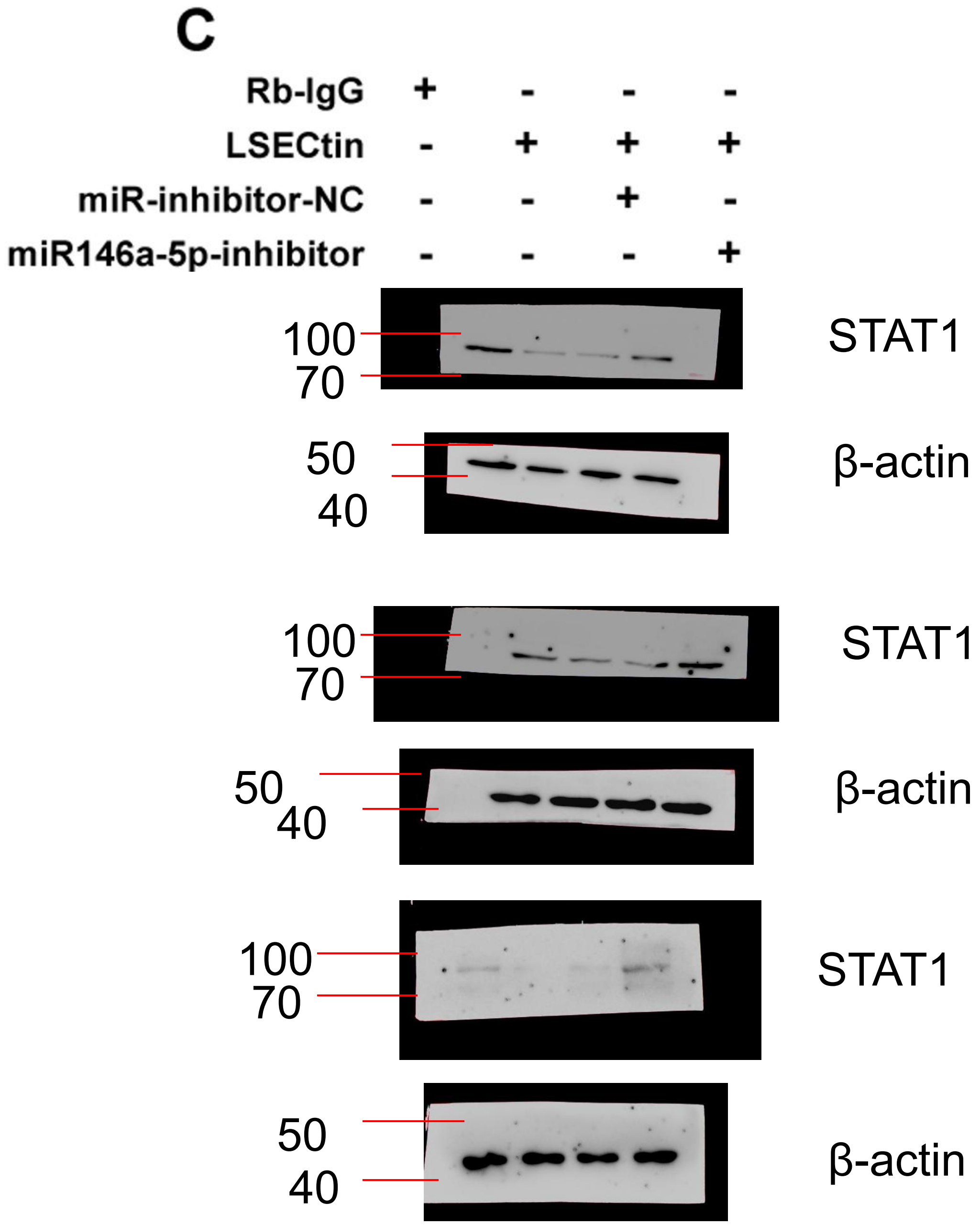
C
Rb-IgG + - - -
LSECtin - + + +
miR-inhibitor-NC - - + -
miR146a-5p-inhibitor - - - +
100
70
STAT1
50
40
β-actin
100
70
STAT1
50
40
β-actin
100
70
STAT1
50
40
β-actin

Fig 6

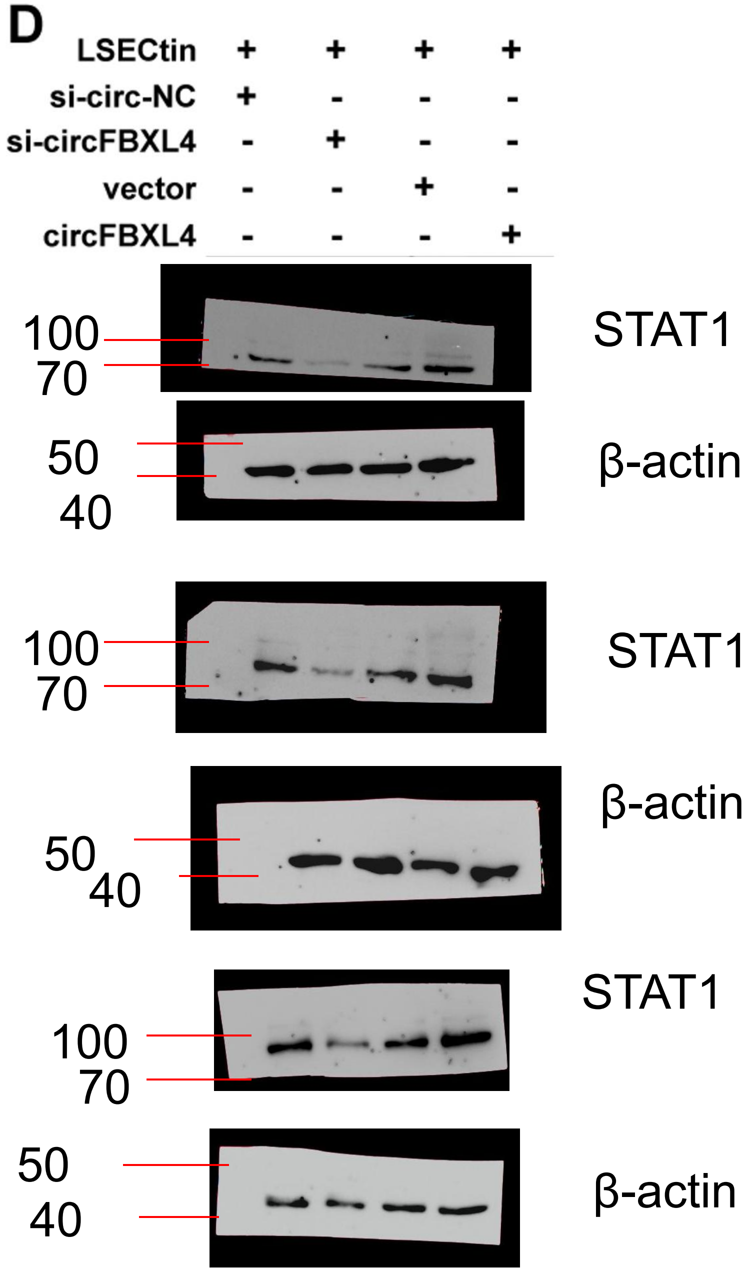
D
LSECtin + + + +
si-circ-NC + - - -
si-circFBXL4 - + - -
vector - - + -
circFBXL4 - - - +
100
70
STAT1
50
40
β-actin
100
70
STAT1
50
40
β-actin
100
70
STAT1
50
40
β-actin

Fig 6

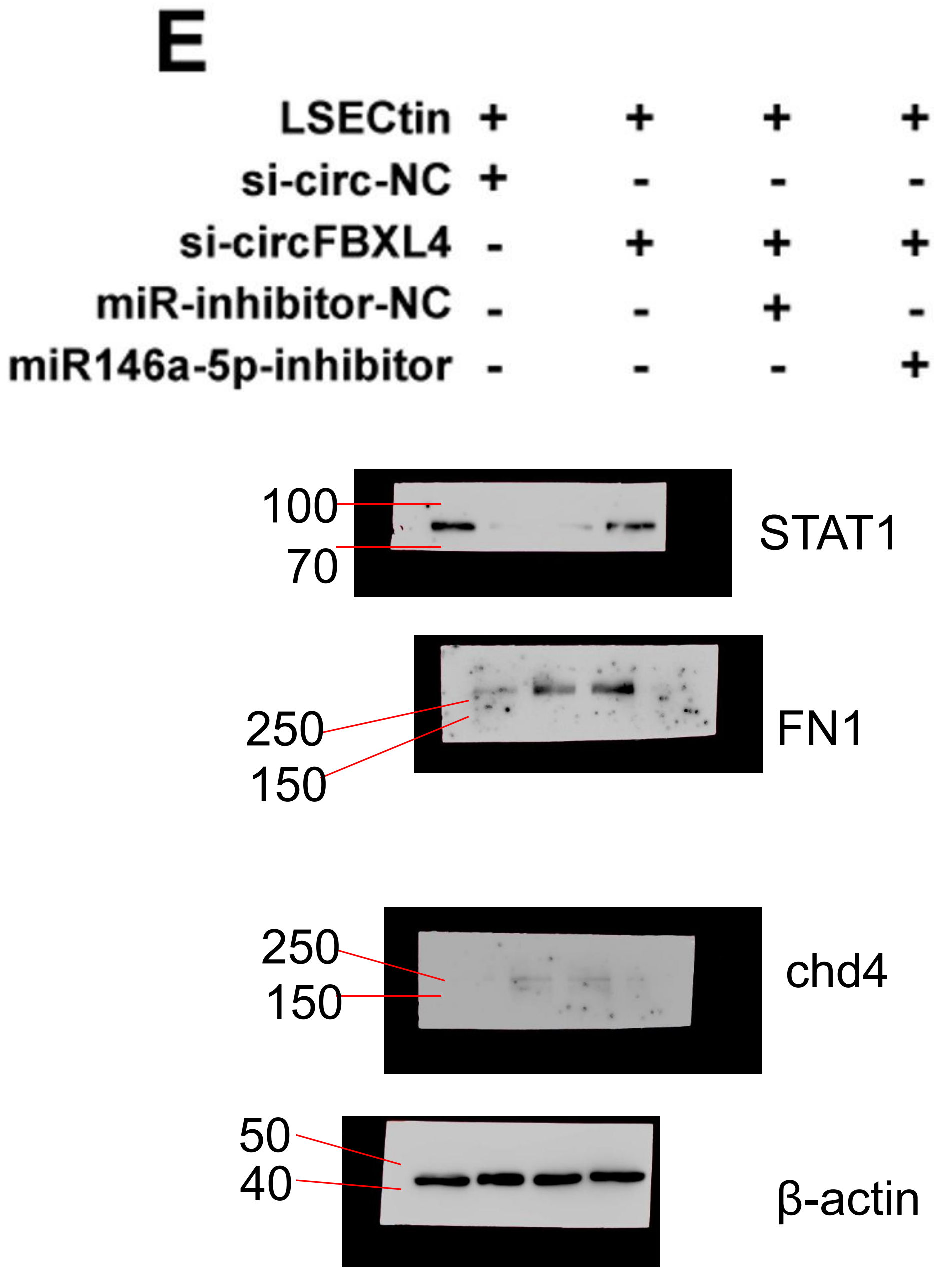
E
LSECtin + + + +
si-circ-NC + - - -
si-circFBXL4 - + + +
miR-inhibitor-NC - - + -
miR146a-5p-inhibitor - - - +
100
70
STAT1
250
150
FN1
250
150
chd4
50
40
β-actin

Fig S2

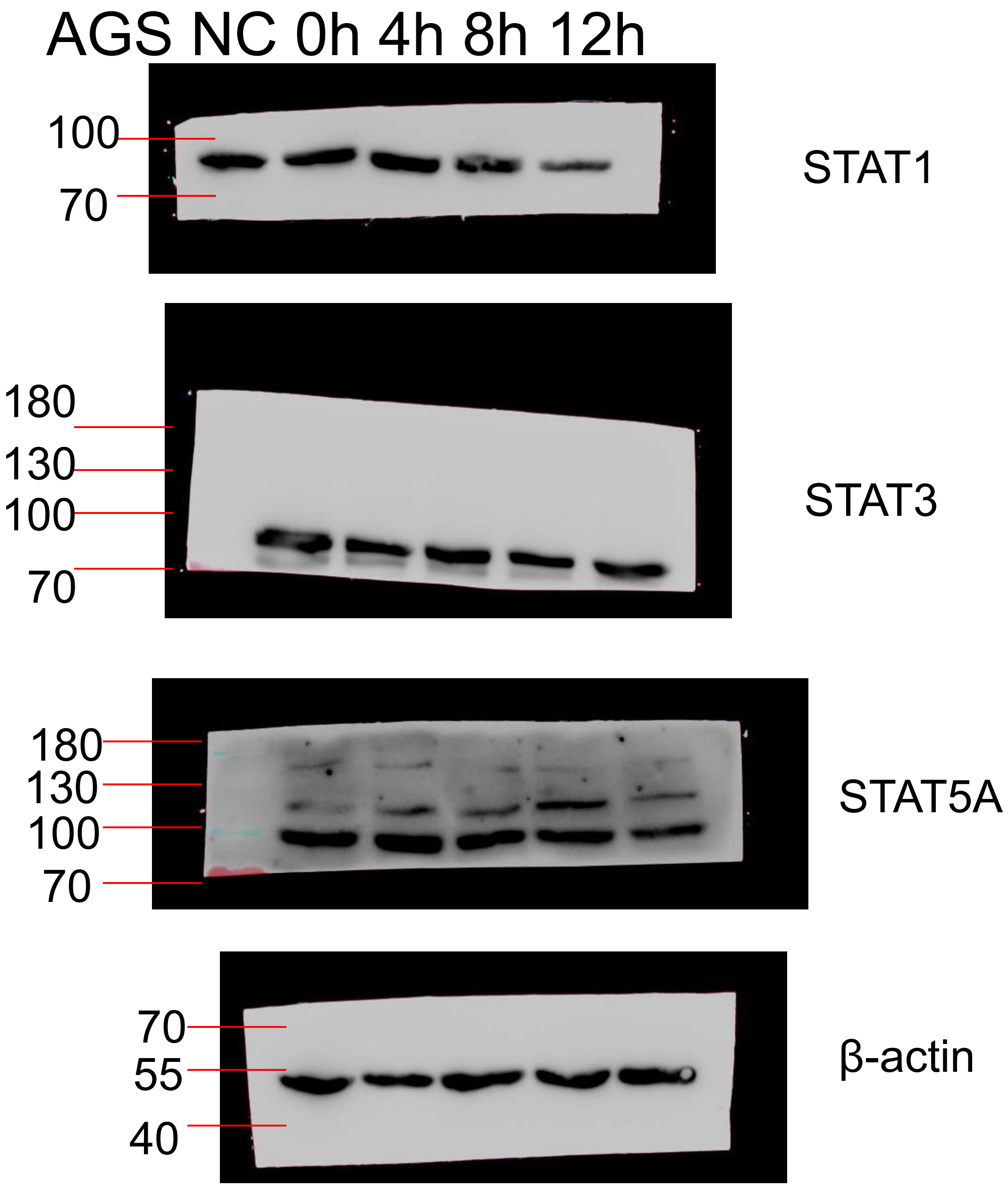
AGS NC 0h 4h 8h 12h
100
70
STAT1
180
130
100
70
STAT3
180
130
100
70
STAT5A
70
55
40
β-actin

Fig S2

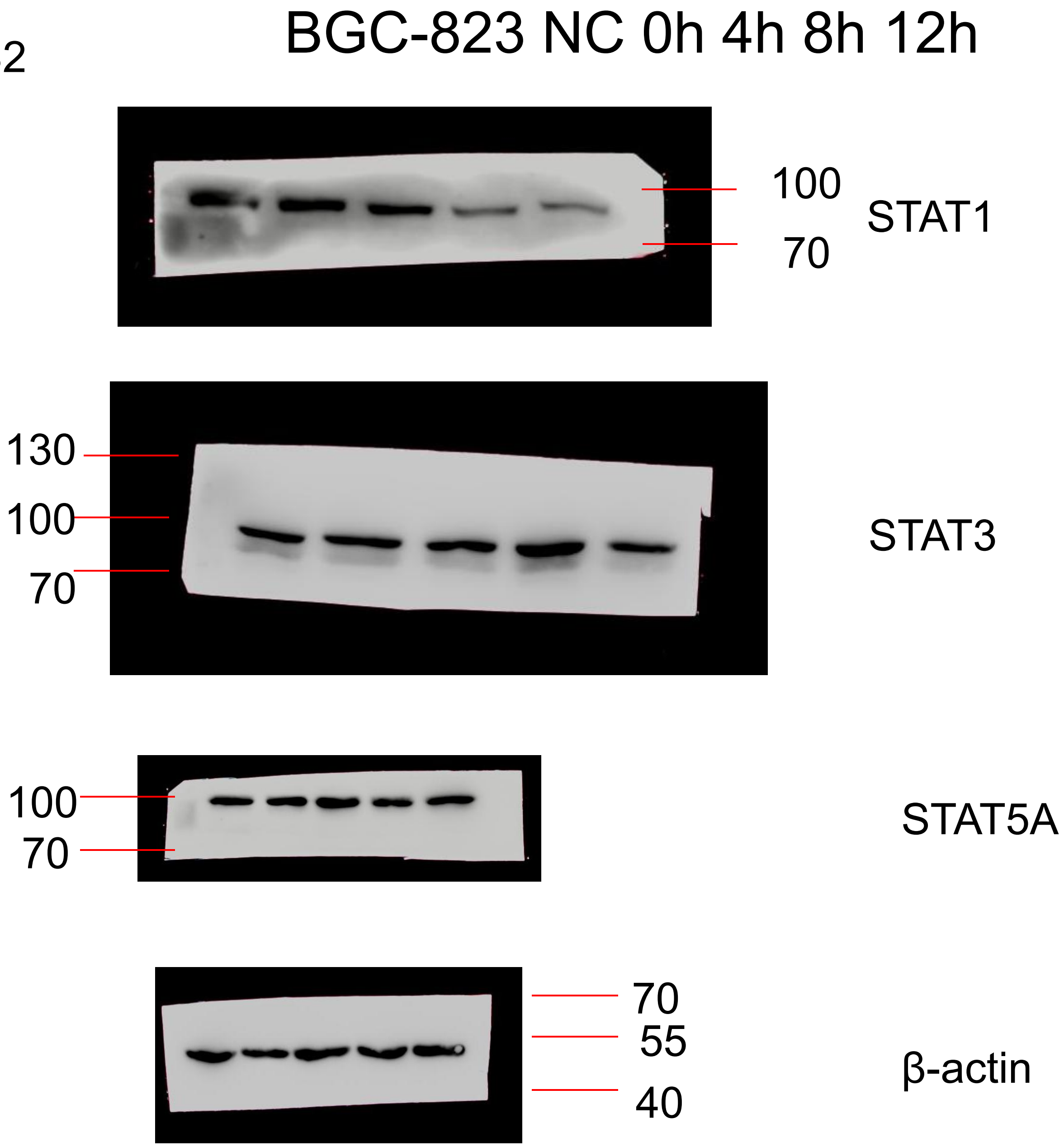
BGC-823 NC 0h 4h 8h 12h
100
70
STAT1
130
100
70
STAT3
100
70
STAT5A
70
55
40
β-actin

Fig S2

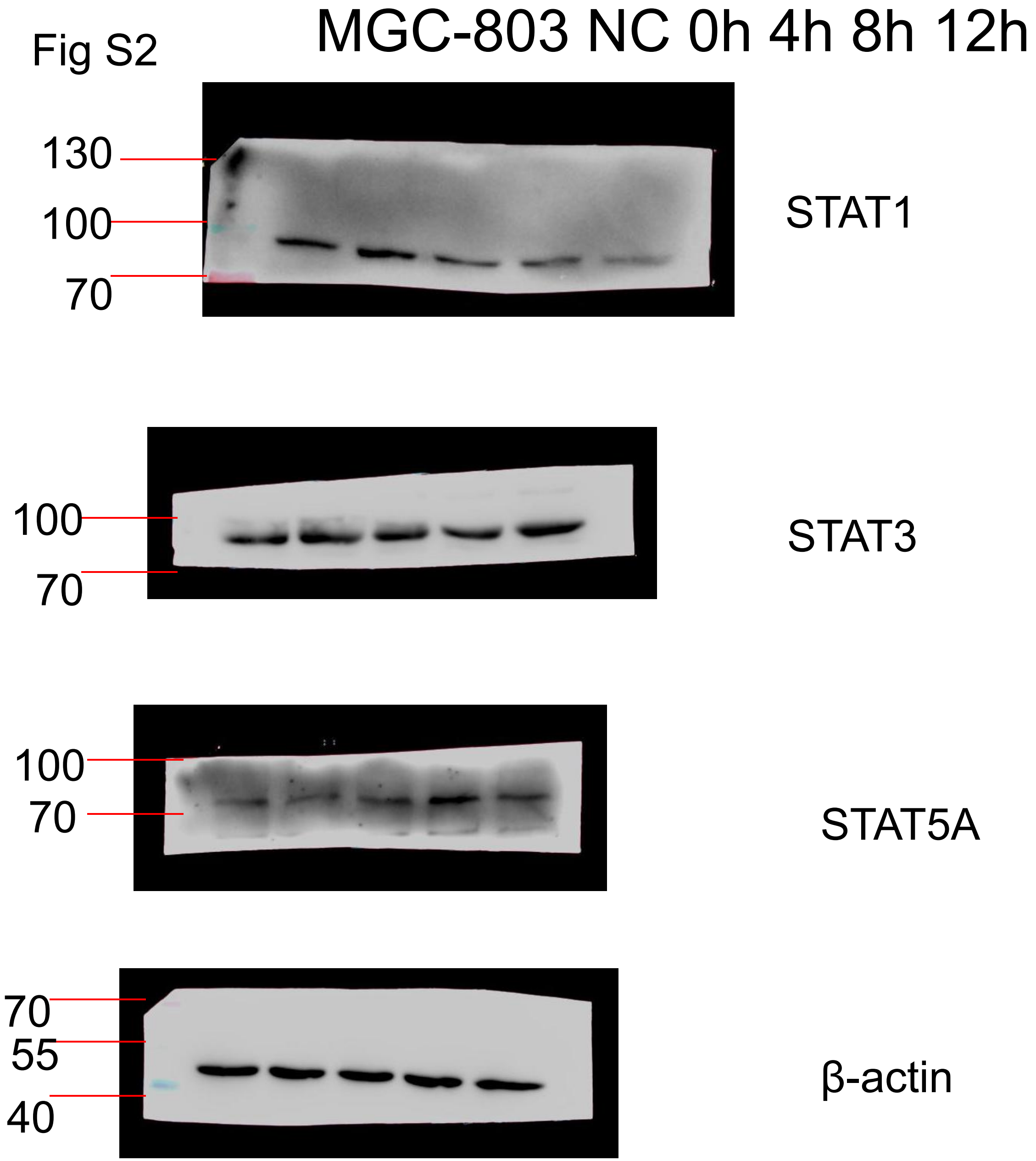
MGC-803 NC 0h 4h 8h 12h
130
100
70
STAT1
100
70
STAT3
100
70
STAT5A
70
55
40
β-actin
